# Transcriptional Profiling of CD8+ CMV-Specific T Cell Functional Subsets Obtained Using a Modified Method for Isolating High-Quality RNA From Fixed and Permeabilized Cells

**DOI:** 10.3389/fimmu.2020.01859

**Published:** 2020-09-02

**Authors:** Zachary R. Healy, Kent J. Weinhold, David M. Murdoch

**Affiliations:** ^1^Division of Pulmonary and Critical Care Medicine, Department of Medicine, Duke University Hospital, Durham, NC, United States; ^2^Department of Surgery, Duke University School of Medicine, Durham, NC, United States

**Keywords:** polyfunctional T cells, CMV, RNAseq, fixation, permeabilization, RNA quality

## Abstract

Previous studies suggest that the presence of antigen-specific polyfunctional T cells is correlated with improved pathogen clearance, disease control, and clinical outcomes; however, the molecular mechanisms responsible for the generation, function, and survival of polyfunctional T cells remain unknown. The study of polyfunctional T cells has been, in part, limited by the need for intracellular cytokine staining (ICS), necessitating fixation and cell membrane permeabilization that leads to unacceptable degradation of RNA. Adopting elements from prior research efforts, we developed and optimized a modified protocol for the isolation of high-quality RNA (i.e., RIN > 7) from primary human T cells following aldehyde-fixation, detergent-based permeabilization, intracellular cytokines staining, and sorting. Additionally, this method also demonstrated utility preserving RNA when staining for transcription factors. This modified protocol utilizes an optimized combination of an RNase inhibitor and high-salt buffer that is cost-effective while maintaining the ability to identify and resolve cell populations for sorting. Overall, this protocol resulted in minimal loss of RNA integrity, quality, and quantity during cytoplasmic staining of cytokines and subsequent flourescence-activated cell sorting. Using this technique, we obtained the transcriptional profiles of functional subsets (i.e., non-functional, monofunctional, bifunctional, polyfunctional) of CMV-specific CD8+T cells. Our analyses demonstrated that these functional subsets are molecularly distinct, and that polyfunctional T cells are uniquely enriched for transcripts involved in viral response, inflammation, cell survival, proliferation, and metabolism when compared to monofunctional cells. Polyfunctional T cells demonstrate reduced activation-induced cell death and increased proliferation after antigen re-challenge. Further *in silico* analysis of transcriptional data suggested a critical role for *STAT5* transcriptional activity in polyfunctional cell activation. Pharmacologic inhibition of *STAT5* was associated with a significant reduction in polyfunctional cell cytokine expression and proliferation, demonstrating the requirement of STAT5 activity not only for proliferation and cell survival, but also cytokine expression. Finally, we confirmed this association between CMV-specific CD8+ polyfunctionality with *STAT5* signaling also exists in immunosuppressed transplant recipients using single cell transcriptomics, indicating that results from this study may translate to this vulnerable patient population. Collectively, these results shed light on the mechanisms governing polyfunctional T cell function and survival and may ultimately inform multiple areas of immunology, including but not limited to the development of new vaccines, CAR-T cell therapies, and adoptive T cell transfer.

## Introduction

High-throughput RNA sequencing (RNAseq) is a powerful tool for the quantification and global analysis of the transcriptome of cells, providing valuable insight to cell function and mechanisms of disease. However, RNAseq requires high-quality RNA obtained from homogeneous cell populations. Unfortunately, immune cell populations are heterogenous, obscuring the signals of rare cell populations such as polyfunctional T cells. The most efficacious method for the isolation of rare populations is fluorescence-activated cell sorting (FACS), which provides the highest cell purity of any technique currently available with minimal bias (1). While high-quality RNA can be obtained from FACS of live cells, investigators are currently limited to isolating cell populations based on surface protein expression. Intracellular targets (e.g., cytokines, transcription factors, phosphoproteins) require cell fixation and permeabilization that leads to unacceptable degradation of RNA. Cell fixation with aldehydes leads to significant RNA-protein crosslinking and fragmentation, while permeabilization with ionic detergents leads to further fragmentation and allows for the introduction of detrimental RNases to the intracellular compartments (2). Ultimately, this processing can lead to significant degradation of RNA quality, as measured by both the RNA integrity number (RIN) and the DV200 (3, 4). Such degradation has been associated with poor or incomplete transcriptional profiling (5). Furthermore, the effects of RNA degradation occurring during fixation and permeabilization are non-uniform and not solely dependent on transcript length, but also other factors including the CG content and 3′ untranscribed region (UTR), potentially introducing further bias (5). Thus, maintaining RNA quality is of utmost importance in generating robust, unbiased RNAseq datasets.

RNA quality is most commonly determined by RIN, a user-independent automated measure of RNA integrity scored on a scale of 1–10, where 1 represents fully degraded RNA and 10 represents fully-intact RNA (4). Cell fixation and permeabilization both contribute to lower RIN values (4). Reduced RNA quality is associated with a loss of complexity, a compromised ability to detect low abundance transcripts, and an inability to detect splice variants (5, 6). Additionally, these studies suggest that the RIN directly correlates with the number of reads successfully mapped to the genome. An alternative measure of RNA quality was introduced by Illumina in 2014, termed DV200 (i.e., the percentage of RNA fragments/molecules greater than 200 bp in length) (3). This measure has been applied to determine the suitability of RNA isolated from formalin-fixed, paraffin-embedded (FFPE) tissues for RNAseq when using a compatible downstream protocol that does not require 3′ polyadenylation or site-specific primers for amplification. This has been effectively used in the literature to qualify RNA from FFPE samples in both limited RNAseq and microarray experiments, where RIN values are commonly very low (<3–5) (3, 7). Generally, a RIN value greater than 7 or a DV200 greater than ~60–70% is suggested as a minimal value for proceeding with library generation.

Several studies have attempted to minimize the impact of fixation and permeabilization on RNA quality, with variable results (8–16). While two recent methods to isolate high-quality RNA from formaldehyde-fixed, saponin-permeabilized, FAC-sorted cell populations have shown promise, each method has potential limitations. The MARIS protocol utilizes commercial RNase inhibitors during each step of processing (i.e., fixation, permeabilization, washing, and staining) and sorting. While effective, the use of RNase inhibitors during each step can become prohibitively expensive depending on the working volumes and number of samples (15). Additionally, RNase inhibitors may only bind specific RNases, and this binding is typically reversible and highly dependent on temperature. Alternatively, the method proposed by Nilsson et al. utilizes a cost-effective high salt buffer (i.e., 2M NaCl) to broadly inactivate RNases (16). While this method is also effective in limiting RNA degradation, the use of a high-ionic buffer can alter antibody tertiary structure and may significantly reduce or alter antibody binding. The resultant decrease in total fluorescence intensity can have dramatic effects on cell population identification and gating strategies, leading to poor population discrimination and erroneous results (17). Therefore, there remain opportunities for further optimization of such techniques.

Growing evidence suggest that antigen-specific polyfunctional T cells play an essential role the immune response to pathogens and malignancy (Supplemental Table S1) (18–25). Polyfunctional cells are defined by the ability to express multiple cytokines (e.g., IFNγ, TNFα, IL-2) and/or chemokines upon antigen re-challenge, and their presence has been associated with improved cytolytic activity, pathogen clearance, and clinical recovery in both human and animal models of infection, vaccination, and malignancy. In contrast, antigen-specific cells expressing only one (i.e., monofunctional) or no cytokines upon antigen encounter have been associated with progressively less cellular protection (21). This phenomenon is perhaps best described in the setting of CMV reactivation following organ transplantation, where recipients are immunosuppressed and at substantial risk for opportunistic infections (21, 24–41). CMV reactivation following organ transplant occurs in 25–40% of recipients, and has been associated with increased episodes of acute and chronic rejection, allograft dysfunction, and early mortality (42). Studies from multiple transplant populations have shown that the presence of CD8+ CMV-specific polyfunctional T cells are associated with a low risk of CMV reactivation in the post-transplant period, whereas a lack of polyfunctional T cells has been associated with a significantly increased risk of reactivation (24–41). Moreover, when CMV reactivation does occur, CD8+ CMV-specific polyfunctional cells have been associated with superior viral control and clearance of viremia (33–35, 39, 40). Recently, Snyder et al. demonstrated not only that CMV-specific CD8+ polyfunctional T cells (IFNγ+/TNFα+/IL-2+/CD107a−) are protective against CMV reactivation, but that the presence of less functional CMV-specific CD8+ subsets (IFNγ+/TNFα−/IL-2-/CD107a+) are detrimental, indicating substantial molecular differences may exists between these antigen-specific cells functional subsets (25). While it is clear that antigen-specific polyfunctionality is an important clinical correlate of T cell efficacy, the mechanism responsible for these differences between polyfunctional and less-functional (i.e., monofunctional) subsets of antigen-specific T cells remains largely unknown. And while recent evidence suggests that antigen-specific CD4+ polyfunctional T cells and their less functional counterparts (i.e., monofunctional cells) are transcriptionally distinct, the mechanism responsible for the generation, survival, and activity remain largely undiscovered (18). Understanding these mechanisms would not only provide novel insights regarding immune responses, but may also lend to the development of vaccines and optimized *ex-vivo* cell expansion protocols for the production of polyfunctional T cells.

To date, the molecular study of antigen-specific polyfunctional T cells has been limited, due in part to their low frequency in peripheral blood, often accounting for less than 0.1% of CD4+ and CD8+ T cell subsets. Additionally, identification of polyfunctional cells requires fixation and permeabilization in order to perform intracellular cytokine staining (ICS), limiting the utility of these samples for downstream assays. With these issues in mind, we therefore sought to develop a modified protocol for the isolation of high-quality RNA from fixed and permeabilized cells that optimizes antibody binding while minimizing overall cost. We then utilized this method to analyze the transcriptome of CMV-specific polyfunctional CMV-specific CD8+T cells from healthy human peripheral blood mononuclear cells (PBMCs). This information was then used to further characterize features unique to polyfunctional T cells, including reduced activation-induced apoptosis and improved proliferation following antigen re-challenge. Additionally, we found that polyfunctional T cells require STAT5, not only for proliferation, but also for cytokine production. Finally, this critical role for STAT5 signaling identified in healthy subjects was also confirmed in immunocompromised solid-organ transplant recipients.

## Materials and Methods

### PBMC Isolation and Cell Culture

For healthy subjects, peripheral whole blood was obtained from Duke IRB-approved (Pro00070584) anonymous donors using ACD vacutainer tubes (BD Biosciences), and PBMCs were isolated using Ficoll density centrifugation (GE HealthCare). PBMCs were counted and viably cryopreserved in LN_2_ vapor (10% DMSO, 90% heat-inactivated FBS). Where appropriate, cells were cultured in RPMI-1640 media containing 10% heat-inactivated FBS (Gibco) and 1x penicillin-streptomycin-glutamine (Gibco) at 37°C and 5% CO_2_. For single cell sequencing in immunosuppressed subjects, cryopreserved PBMC samples from two recipients were obtained from the Duke IRB-approved Abdominal Transplant Repository (ATR) (Pro00035555). Kidney, liver, pancreas, and small intestine transplant recipients were recruited prospectively through the Abdominal Transplant clinic at Duke University Hospital and PBMC samples were collected longitudinally at pre-specified time points prior to and following transplantation. For mechanistic CMV reactivation experiments, one subject with and one matched control without CMV reactivation in the first 12 months following transplant were selected. The subjects were matched by age (50–55), HLA-^*^A0201 status (necessary for tetramer use; note: no other matching alleles were required), type of transplant (kidney), induction immunosuppression (none), donor-recipient CMV status (D-/R+), maintenance immunosuppression [prednisone, mycophenolate (MMF), and tacrolimus (FK506)], and CMV prophylaxis (none). PBMC samples were selected from the time point just prior to when CMV reactivation occurred in the case subject (i.e., 3 months post-transplant for both the case and control subject). For determination of the immunophenotype of dextramer+ CMV-specific T cells (Supplemental Figure S11C), data was obtained from pre-transplant PBMC samples of 5 kidney transplant recipients enrolled in the ATR (Pro00035555).

### Peptide and/or Antibody Stimulation

For cell sorting experiments, cell stimulation was performed using pp65 and IE-1 CMV overlapping peptide (JPT Laboratories). Human PBMCs were stimulated with both pp65 and IE-1 overlapping peptides (1 μg/μL final concentration of each peptide) in R10 at a cell concentration of 2 x 10^7^ cells/mL for 6 h. The final concentration of DMSO was less than 0.2%. CD107a-PE was included during stimulation. Cells were stimulated in the presence of brefeldin A (BFA) and monensin per manufacturer protocol (BD Bioscience) for the final 4 h prior to surface staining. For experiments involving the use of dextramers for stimulation, stimulation was performed by incubating cells with the dextramer and costimulatory antibodies (αCD28, αCD49; BD Biosciences) for 6 h, with BFA and monensin added for the final 4 h of the stimulation.

### Antibodies

All antibodies for flow cytometry were monoclonal antibodies and were purchased from BD Biosciences, Biolegend, ThermoFisher (previously eBiosciences), or RnD Systems. All antibodies were titrated to optimal signal-to-noise ratio on T cells prior to use, assuming a 50 mL staining volume. Cell viability was determined using Zombie Violet Fixable Viability Kit (0.1 μL per 50 μL staining volume; Biolegend) or Zombie-NIR Fixable Viability Dye. Antibodies used for sorting the functional subsets was as follows: CD14-BV510 (M5E2 clone), CD3-BV785 (SK7 clone), CD4-FITC (SK3 clone), CD8-APC-Cy7 (HIT8a clone), IFNγ-PE-Cy7 (4S.B3 clone), TNFα-AF700 (MAb11 clone), IL-2-APC (MQ1-17H12 clone), and CD107a-PE or CD107a-PE-Cy5 (H4A3 clone). Additional antibodies used for immunophenotyping include the following: CD3-BUV395 (SK7 clone), CD4-BV605 (SK3 clone), CD8-BUV805 (HIT8a clone), IL-4-BV711 (Rat MP4-25D2 clone), IL-17-BV421 (BL168 clone), Perforin-BV711 (dG9 clone), KLRG1-BV421 or KLRG1-AF647 (SA231A2 clone), CD127-BV650 (A019D5 clone), CCR7-BB700 and CCR7-BV785 (3D12 clone), CD45RA-BB515 (HI100 clone), PD-1-BV711 (EH12.2H7 clone), CD107a-PE-Cy5, Annexin V-BV711, CD95-BV786 (DX2 clone), active Caspase-3-AF647 (C92-605 clone), CD25-BV421 (clone M-A251), CD69-APC-Cy7 (clone FN50), CD137-APC (clone 4B4-1), CD154-APC (clone 5C3), and TNFSF8-PE (CD30L;clone MAB1028, RnD Systems). Proliferation was monitored using proliferation dye Tagit-Violet (Biolegend). CMV-specific MHC I Dextramers (pp65-A^*^0201) labeled with PE were purchased from Immudex (Fairfax, VA, USA). For cytokine secretion assays, the following were purchased from Miltenyi Biotec: IFNγ-FITC kit (no specified clones), TNFα-PE, IL-2-APC. These assay kits were used according to manufacturer protocol assuming less than 5% of the cell population will express the desired antigen. When used in tandem, the concentration of each antibody was maintained at the manufacturer-recommended amount of 10 μL per 100 μL staining reaction, with an equivalent reduction in the base staining buffer (PBS with 2% HI-FBS) to maintain the appropriate volume.

### Surface and Intracellular Staining

First, CD8+ T cells were negatively enriched by magnetic separation (ThermoFisher), counted, and after adjusting for viability resuspended at 10 x 10^6^ cells/mL. After washing the cells twice with 1xPBS containing 1% heat-inactivated (HI)-FBS, surface staining was performed at 4°C for 25 min in the dark. Following surface staining, the cells were washed three times in wash buffer (1xPBS with 1% HI-FBS) and then subjected to fixation and permeabilization steps as per manufacturer or published protocol. For the modified protocol, cells were fixed and permeabilized in a solution containing 4% PFA, 0.1% saponin, and 1 unit/μL of RNasin Plus RNase inhibitor (Promega) on ice for 20 min in dark. Cells were then washed twice in permeabilization buffer (0.1% saponin, 2M NaCl, 0.5% BSA in RNase-free PBS), and intracellular staining was performed in a permeabilization-stain buffer (1x PBS with 0.1% saponin containing 1 unit/μL of RNasin Plus RNase inhibitor as well as the desired antibody) at 4°C for 30 min. Intracellular staining was performed at 10 x 10^6^ cells/mL. The cells were then washed twice in permeabilization buffer, and resuspended in sort buffer (1% BSA, 2M NaCl in RNase-free PBS). For BD CytoFix/CytoPerm, Biolegend TrueNuclear, and eBioscience FoxP3 buffer kits, all staining was performed according to manufacturer protocol. For STAT5-BV421 (Y694) phosphoprotein staining, BD buffer III was used according to manufacturer protocol following initial intracellular staining for cytokines with methanol-resistant fluorophores. Surface staining for phenotypic markers was performed following phospho-protein staining.

### Fluorescence-Activated Cell Sorting (FACS)

To obtain all necessary cell populations of interest, five-way fluorescence-activated cell sorting was performed using a Duke Human Vaccine Institute (DHVI) BD Influx instrument with a 70 μm nozzle, producing a drop size of ~1.5 nL/cell. Cold blocks were used to maintain the sort sample and collection samples at 4°C, and the sample was collected into RNase-free 1.5 mL tubes containing 500 μL of collection buffer (1% BSA, 4M NaCl in RNase-free PBS) to account for dilution. A maximum of 500 μL of cells were sorting into each tube to prevent dilution below 2M NaCl. When the Influx instrument was not available, 4-way sorting was performed using BD Aria II cell sorter in a similar manner. In this case, the negative population was isolated first (***pop A***), followed by four-way sorting to obtain ***pop. B-E*.** Immunophenotyping experiments were performed using a BD LSRFortessa, X20, a BD Aria II, or a Cytek Northern Lights spectral flow cytometer. All instruments used in these experiments undergo routine preventative maintenance and quality control to ensure optimal functioning.

### Quantification of Proliferation, Cell Survival, Surface Marker, and Cytokine Expression Within Functional Subsets of CMV-Specific T Cells

For these experiments, cells were first stained with Tagit-Violet proliferation dye (Biolegend) according to manufacturer protocol, stimulated for 24 h with overlapping pp65 and IE-1 peptide (1 μg/mL), and then CMV peptide was removed and the cells were incubated in R10 for an additional 5 days (6 day total). On day 3, cells were supplemented with IL-2 (10 U/mL). Cells were then re-stimulated with overlapping CMV peptide for 12 h and stained for viability (Zombie dye; Biolegend), phenotype (CD3, CD4, CD8), type 1 cytokines (IFNγ, TNFα, IL-2) in addition to other cytokines (IL-4, IL-17), degranulation marker CD107a, cytotoxic molecules (Granzyme B, Perforin), cell surface markers (CCR7, CD45RA, CD127, KLRG1, PD-1), and markers of apoptosis (Annexin-V, CD95, active-caspase-3). This method of sequential CMV stimulation had the beneficial effect of reducing the number of naïve and non-CMV-specific T cells that were present in the non-functional and to a lesser extent the IFNγ monofunctional cell populations. For proliferation, the percentage of cells proliferating and the division index were then determined within each functional subset. For cell survival, viability, and surface phenotype staining was performed first, followed by Annexin-V staining in a calcium-containing PBS buffer provided by the manufacturer and according to manufacturer protocol (Biolegend). Cells were then washed, fixed and permeabilized to allow for intracellular staining of cytokines and active caspase-3. Populations were defined as follows: Early Apoptotic: Zombie-/Annexin-V+; Late Apoptotic: Zombie+/Annexin-V+; Necrotic: Zombie+/Annexin-V-; Surviving:Zombie-/Annexin-V-.

### Chemical Inhibitors

For chemical inhibition experiments, all reagents were purchased from Calbiochem (EMD-Millipore/Sigma; Burlington, MA). Cells were incubated for 2 h in indicated concentration of inhibitor prior to CMV stimulation, and the concentration of inhibitor was maintained throughout stimulation.

### RNA Isolation and RNA Quality

Cells were isolated from sort buffer by centrifugation at 2000 RCF for 10 min at 4°C. The increase in centrifugation speed during this post-processing step was necessary to improve yield, as the 2M buffer is more viscous than a standard PBS buffer (137 mM) at low temperature. Total RNA was then isolated per protocol using the Ambion FFPE RNA isolation kit starting at the protease digestion step, starting at the step following the xylene removal. Additionally, the proteinase K step was modified from the recommended 15 min at 50°C followed by 15 min at 80°C to a single 60-min step at 50°C, as this modestly improved the RNA quality. Isolated total RNA was then submitted to the Genomic and Computation Biology (GCB) Core Facility at Duke University for RNA quantification and quality check using the Agilent 2100 Bioanalyzer and a PICO chip. For live cell RNA isolation, the Qiagen RNeasy Mini kit was used per manufacturer protocol.

### Total RNA Library Preparation and Sequencing

Library preparation was carried out per manufacturer protocol using the Clontech PICO-input total RNA library kit, using 0.5–1.0 ng of total RNA input. Due to the partially degraded nature of the RNA, the RNA fragmentation step was reduced from 4 to 2 min. Resultant cDNA libraries were submitted to the Duke GCB Core for DNA quality check using the Agilent 2100 Bioanalyzer. The libraries were then submitted to the Sequencing Core for library quantification and pooling. Sequencing was performed using an Agilent HiSeq 4000 instrument (2 x 150 bp). As these experiments utilized the first generation of the PICO-input total RNA library kit, this required a 20% PhiX spike-in to alleviate issues with low complexity of SMARTer stranded kits in the first three cycles. Pooling was performed to allow for 50–60 million reads per sample. The five libraries for each individual were randomized between the three sequencing lanes to avoid batch-effect issues.

### RNASeq Processing and Analysis

RNA-seq data was processed using the TrimGalore toolkit which employs Cutadapt to trim low quality bases and Illumina sequencing adapters from the 3′ end of the reads. Only reads that were 20 nt or longer after trimming were kept for further analysis. Reads were mapped to the GRCh37v73 version of the human genome and transcriptome using the STAR RNA-seq alignment tool. Reads were kept for subsequent analysis if they mapped to a single genomic location. Gene counts were compiled using the HTSeq tool. Only genes that had at least 10 reads in any given library were used in subsequent analysis. Normalization and differential expression was carried out using the DESeq2 Bioconductor package within the R statistical programming environment. The false discovery rate was calculated to control for multiple hypothesis testing. RUVseq was used to determine potential batch effects. Gene set enrichment analysis, KEGG analysis, and Pathview were performed to identify differentially regulated pathways and gene ontology terms for each of the comparisons performed. All subsequent secondary analysis was performed in R or using the Ingenuity Pathway Analysis tool (Qiagen) through the Duke CGCB Analysis Core.

### BD Precise Single-Cell Sequencing

All steps were performed according to manufacturer protocol (BD Single Cell WTA Precise Assay V2). Briefly, following dextramer-based stimulation, cells from the two transplant recipients (248, case subject with CMV reactivation; 249, control without CMV reactivation) were surface-stained for phenotypic and maturation markers, and 96-individual CMV-specific CD8+ cells (viability-/CD14-/CD3+/CD8+/pp65-A^*^0201+) were sorted into individual wells of a 96-well plate and flash-frozen using an EtOH-dry ice bath and stored at −80°C. Libraries were prepared according to manufacturer protocol, and library quality and quantity determined using an Agilent 2100 Bioanalyzer. Sequencing was performed by the Duke CGCB Sequencing Core using the Agilent MiSeq V3 platform (2 x 75 bp), and analysis was performed using the BD Precise WTA Analysis Pipeline v2.0 on the Seven Bridges Platform according to manufacturer protocol (BD Precise WTA Analysis Users Guide). The DBEC Molecular Index Counts were used for analysis and visualization in BD DataView v1.2.2.

### Statistical Analysis of RNA Quantity and Quality Data

All flow cytometry analysis was performed in FlowJo v9.9. SPICE software v6.0 was used to generate plots for functional subset distributions. All statistical analysis was performed using Prism GraphPad (version 7). All RNA quantification and quality data was assumed to be parametric. Grouped analysis was performed where appropriate. Statistical significance throughout the manuscript is indicated by the following: ^ϕ^*p* < 0.005; ^*^p < 0.05.

## Results

### Evaluation of Methods for the Isolation of High-Quality RNA From Aldehyde-Fixed and Saponin-Permeabilized Primary Human T Cells Following Intracellular Cytokine Staining

We first examined the efficacy of maintaining high-quality RNA via three protocols: (1) a standard intracellular staining (ICS) protocol, (2) the MARIS protocol, and (3) a high-salt buffer (2M NaCl) protocol. Ficoll density-isolated PBMCs underwent stimulation with CMV peptide, immunomagnetic CD8+ enrichment formaldehyde-fixation, saponin-permeabilization, and intracellular antibody staining (Supplemental Figure S1). Following staining, samples underwent a 2-h hold at 8°C to replicate the time necessary to transport the samples and set-up and perform a FACS isolation. While significant RNA degradation was observed using a standard ICS protocol (Supplemental Figure S1A), both the MARIS and high-salt buffer protocols were effective at maintaining RNA quality during staining. However, we observed variability in RNA quality from the MARIS protocol when samples were held for 2 h to replicate the time necessary to transfer samples and set-up and complete fluorescent-activated cell sorting. In contrast, the high salt buffer protocol demonstrated less variability when compared to the MARIS protocol (mean ± S.D.: Salt Buffer, 6.8 ± 0.1; MARIS 4.9 ± 1.1). Additionally, the MARIS protocol required a significant quantity RNase inhibitor per sample, particularly for washing steps and during sorting. Although the high salt buffer resulted in a slight improvement in RNA stability and was far more cost-effective when compared to the MARIS protocol, prior research has suggested that higher salt concentrations were expected to impact not only antibody binding but also downstream enzymatic processes (e.g., reverse transcription) (17). We, therefore, set out to determine the minimum salt concentration necessary to preserve RNA quality. As the salt concentration increased from that of standard phosphate buffered-solution (PBS, 0.137 M), the RNA quality increased until a concentration of 2M was reached (Supplemental Figure S1B), after which no additional benefit was observed (*data not shown*). Concentrations above 2M resulted in increased sorting stream dispersion due to ionic effects and, therefore, were not tolerable. Unfortunately, the presence of a 2M NaCl buffer during intracellular staining steps significant reduced the staining of several cytokines (i.e., TNFα, IL-2) when compared to a standard protocol, and appeared to have heterogenous effects on the staining of transcription factors (Supplemental Figures S1C,D). This effect appeared to be mostly dependent on the monoclonal antibody clone rather than on the specific fluorophore selected. Importantly, this reduction in antibody signal was not observed with the use of the MARIS protocol (*not shown*). Given the above potential limitations of both the MARIS and high-salt protocols, we developed a modified protocol that utilized the advantages of each technique. The modified protocol utilizes a 2M NaCl buffer during wash and sorting steps instead of RNase inhibitor whose performance in preserving the RIN is variable and quite costly. Instead, an RNase inhibitor is used during the permeabilization and the intracellular staining steps where volumes are minimal. This modified protocol yielded no significant reduction in cytokine or transcription factor staining on the limited number of intracellular targets selected (IFNγ, TNFα, IL-2, CD107a, Tbet, EOMES), indicating that the effect of the high salt buffer is largely due to effects during initial antibody binding (Supplemental Figures S1C,D). Note that this list is not exhaustive, and each intracellular target should be tested for potential interference during titration.

### Effect of a Modified Protocol on RNA Quality Following Staining for Cytokines and Transcription Factors

**Figure 1:**
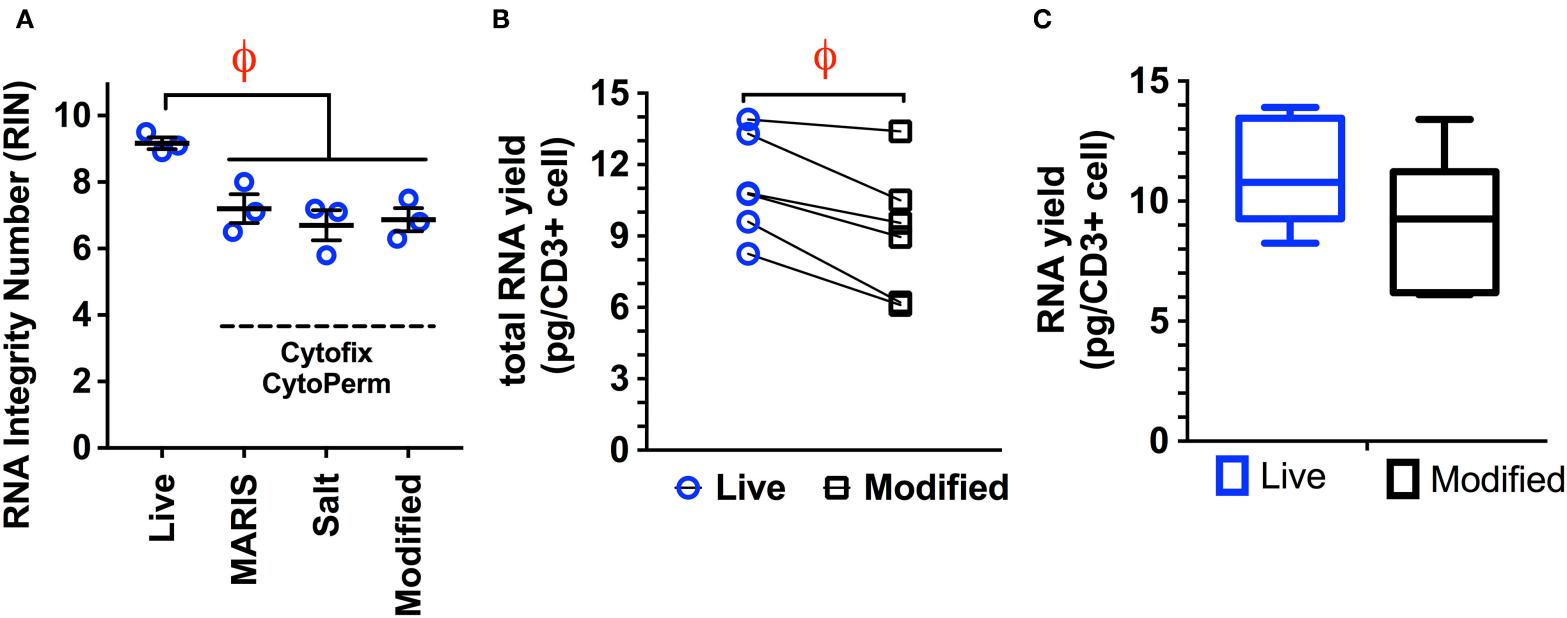
**(A)** Comparison of RNA integrity numbers (RIN) from live cells and cells isolated using the MARIS, High Salt (2M NaCl), and modified protocol for the isolation of RNA from fixed and permeabilized samples. Data is represented as the RNA integrity number (RIN) following all processing, staining, and sorting steps (viable, CD14–, CD3+; *n* = 3 per condition). While there is a significant reduction in RIN from live cells to those isolated following all three of the above protocols, there is no significant difference between either of the three protocols with regard to RNA quality, with all three producing RNA with acceptable quality. **(B,C)**. RNA yield obtained using the modified protocol demonstrating a small but significant reduction (18%) in total RNA yield when comparing live cells and those processed using the modified protocol (*n* = 5). This mild reduction in RNA quantity would have a minimal impact on the number of cells required to generate adequate RNA libraries. Statistical testing: ^ϕ^*p* < 0.005.

We next examined the ability of our modified protocol to prevent RNA degradation using commercial kits for both cytokine (*BD Cytofix/Cytoperm*) and transcription factor staining (*Biolegend TrueNuclear, eBioscience FoxP3*). The modified protocol proved equivalent to both the MARIS and high salt protocols when a standard formaldehyde/saponin buffer was used, as measured by the RIN (mean ± S.D: MARIS, 7.2 ± 0.43; high salt, 6.7 ± 0.45; modified 6.9 ± 0.35; Figure 1A). This method was associated with only an 18% reduction in total RNA yield when compared to RNA isolation from live cells (Figures 1B,C), with an average yield of 9.12 pg per cell. This small reduction in RNA quality would have minimal impact on the number of sorted cells required to generate sequencing libraries. Therefore, when staining for intracellular cytokines, this modified protocol yields RNA with sufficient quality and quantity to generate bulk sequencing libraries from populations as small as 100 cells.

We next examined the effect of our protocol on maintaining RNA integrity when performing staining for nuclear targets. Nuclear targets, such as Helios to identify T regulatory cells, are often of interest in identifying specific immune cell subpopulations. While staining for some nuclear targets can be accomplished with more mild detergent concentrations, optimal staining requires the use of much harsher conditions. Under these more rugged conditions, we found that even with this optimized protocol, RIN values were reduced when using commercially available buffers for nuclear staining (Supplemental Figures S2A,B). However, the modified protocol is able to maintain the DV200 above 60%. Overall, the yield of RNA from samples treated with nuclear permeabilization reagents was not significantly different than that of more mild agents.

### Isolation of Functional Subsets of CMV-Specific T Cells and Generation of Stranded, Total RNAseq Libraries Using the Modified Protocol

**Figure 2:**
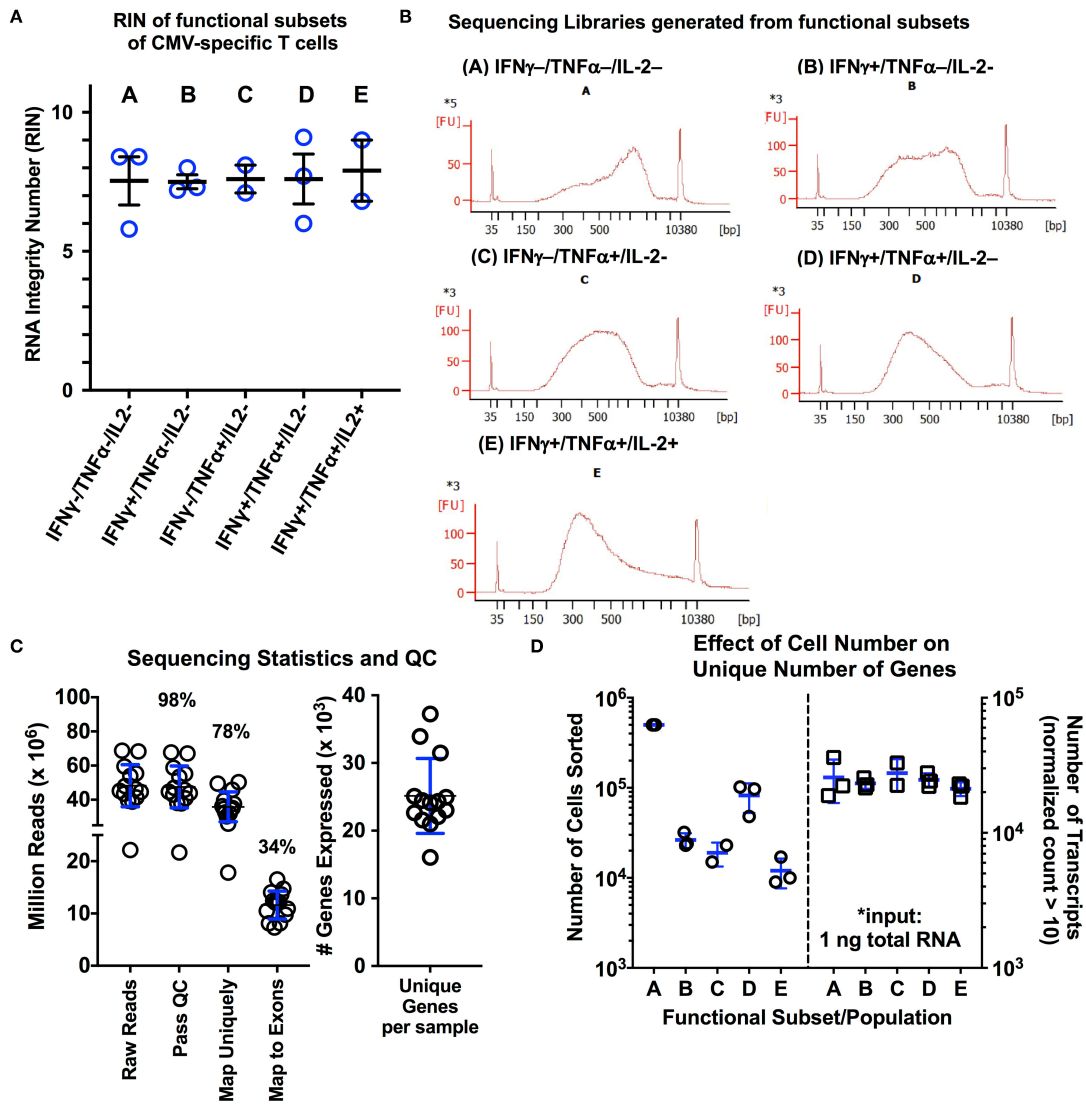
**(A,B)** RNA quality measurements and representative cDNA libraries for functional subsets CMV-specific CD8+ T cells isolated using the modified protocol. Following activation with a CMV peptide pool, cells were processed and stained using the modified protocol, and the five predominant functional subsets of CMV-specific CD8+ T cells were sorted. Following RNA isolation using the Ambion FFPE Recoverall kit, RIN values were obtained for three different donors (*n* = 3 subjects; blue lines on graph represent mean ± SD). Of the 15 individual RNA samples, two of the RNA electropherograms were unable to be fitted by the RIN algorithm due to the low overall RNA input. The average RIN value of the remaining 13 libraries was 7.60 ± 1.03. **(B)** Representative cDNA libraries generated using the Clontech PICO input stranded total RNA kit are shown. **(C)** Sequencing statistics and quality control analysis results for the 15 libraries following sequencing on the Illumina HiSeq4000 platform (2 x 150 bp; 20% PhiX spike-in; 5 samples per lane). The average number of unique transcripts detected was 25.127 ± 5.53 (x10^3^) per sample (blue lines on graph represent mean ± SD). **(D)** Examination of the effect of the number of sorted cells on number of unique transcripts, as a measure of RNA complexity (*n* = 3; blue lines on graph represent mean ± SD). A total of 1 ng of total RNA was used for library generation for each population, independent of the number of cells collected during sorting. Overall, we observed no significant effect of cell number isolated during sorting on the number of unique transcripts. Statistical testing: *p < 0.05. Populations: **(A)** non-functional (IFNγ-/TNFα-/IL-2-); **(B)** IFNγ only/monofunctional (IFNγ+/TNFα-/IL-2-); **(C)** TNFα monofunctional (IFNγ-/TNFα+/IL-2-); **(D)** bifunctional (IFNγ+/TNFα+/IL-2-); and **(E)** polyfunctional (IFNγ+/TNFα+/IL-2+).

To determine whether our optimized protocol could reliably obtain high quality RNA from relevant clinical samples, we applied our protocol to isolate subpopulations of CMV-specific CD8+ T cells from normal, otherwise healthy subjects (*n* = 3). Following stimulation with pp65 and IE-1 overlapping peptide pools and using a standardized gating protocol, we examined the five predominant CMV-specific CD8+ functional subsets from three normal donors with known CMV reactivity and previously-quantified functional subsets (Supplemental Figures S3A–C). Based on the study by Snyder et al., we chose to include type 1 cytokines IFNγ, TNFα, and IL-2 and the degranulation marker CD107a (LAMP1) in our sorting panel. Due to technical limitations in cellular sorting (i.e., 6-way sorting, 8 drill-down gates), we elected to sort solely based on the type 1 cytokines, but retained CD107a in our sorting panel for analysis purposes. We found that using this approach, there were five predominant functional subsets of CMV-specific T cells: **(A)** non-functional (IFNγ-/TNFα-/IL-2-); **(B)** IFNγ only/monofunctional (IFNγ+/TNFα-/IL-2-); **(C)** TNFα monofunctional (IFNγ-/TNFα+/IL-2-); **(D)** bifunctional (IFNγ+/TNFα+/IL-2-); and **(E)** polyfunctional (IFNγ+/TNFα+/IL2+). We performed a total of two sorting experiments per donor using the above protocol to sort each of the five CD8+ T cell populations. Additionally, we examined markers of T cell differentiation (CCR7, CD45RA) and antigen experience (CD127, KLRG1, PD1). The following observations are important to note: (1) the non-functional subset (***population A***) consisted of cells from each maturational subset (Supplemental Figure S3D), which includes many non-CMV-specific T cells; (2) the IFNγ monofunctional subset (***population B***) did contain variable quantities of naïve T cells, ranging from 4 to 22% across the three donors used; (3) the remaining three subsets (***populations C–E***) showed minimal variability within an individual with a predominance of effector memory (CCR7^−^/CD45RA^−/int^) cells with a CD127^low^KLRG1^hi^ phenotype (Supplemental Figure S3D). Overall, this yielded 5 libraries per subject per experiment. The experiment with the highest RNA yield per donor was used for library preparation with the Clontech Pico Input Mammalian stranded total RNA kit (v1). The RIN values and representative cDNA library electropherograms are shown in Figures 2A,B for each of the five populations. The RIN values for all samples collected was above 5, with an average value of 7.10 ± 1.03; additionally, in all cases, the DV200 was maintained above 68% (Figure 2A). Quality analysis demonstrated performance similar to RNAseq protocols using live cells, with 98% of reads passing initial quality control, 78% mapping uniquely to the human genome, 34% mapping to exons, and ~25,000 unique genes expressed (defined as greater than or equal to 10 reads/sample; Figure 2C) (43). One of the 15 sequencing samples (donor 3, population C, IFNγ-/TNFα+/IL-2-) was identified as an outlier in quality control analysis and was eliminated from further analysis (additional exploratory analysis and visualization data is provided in Supplemental Figures S4–S7). Interestingly, despite the fact that the number of cells isolated from each of the functional subsets ranged from 500,000 cells down to 9,000 cells, there was no significant effect on the number of unique transcripts detected per sample (Figure 2D). This is in line with a recent study showing that Clontech SMART preparation methods showed sensitivity for differentially expressed genes as well as consistency between technical replicates down to an input of ~1,000 activated T cells (43).

### Confirmation of Transcriptional and Phenotypic Differences Between Functional Subsets of CD8+ CMV-Specific T Cells

**Figure 3:**
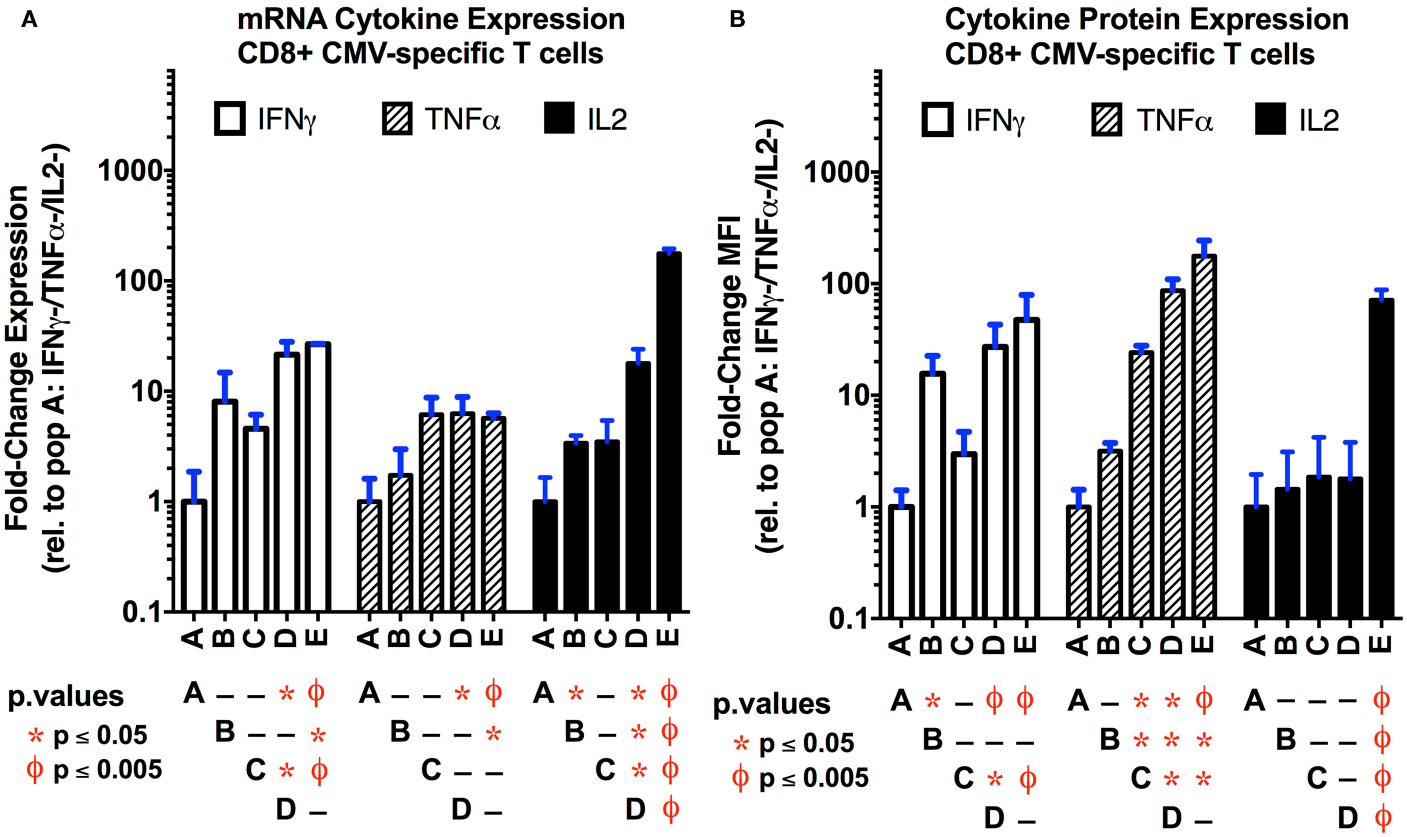
Comparison of cytokine mRNA **(A)** and protein **(B)** expression levels in the functional subsets of CMV-specific CD8+ T cells. The mRNA transcript levels for the three primary type 1 cytokines (IFNγ, TNFα, IL-2) were obtained as normalized counts from total RNAseq data, and expressed as the fold-change (log_2_) compared to the non-functional population (**A**; IFNγ-/TNFα-/IL-2-). The protein expression level **(B)** was obtained by flow cytometry following intracellular staining for the three cytokines of interest. Data are expressed as the fold-change in mean fluorescence intensity (MFI) relative to the non-functional population (*n* = 3 per subset; error bars represent SD). Overall, there is good correlation between the mRNA and protein expression levels. This suggest that, in single cell experiments, the transcription data may be useful to identify functional subsets. Statistical testing: ^ϕ^*p* < 0.005; *p < 0.05. Populations: **(A)** non-functional (IFNγ-/TNFα-/IL-2-); **(B)** IFNγ only/monofunctional (IFNγ+/TNFα-/IL-2-); **(C)** TNFα monofunctional (IFNγ-/TNFα+/IL-2-); **(D)** bifunctional (IFNγ+/TNFα+/IL-2-); and **(E)** polyfunctional (IFNγ+/TNFα+/IL-2+).

In order to establish if transcriptional differences exist between the functional subsets of CMV-specific CD8+ T cells, we compared their transcriptional profiles obtained by total RNAseq. Differentially-expressed gene (DEG) analysis using DESeq2 for coding and long non-coding RNA is shown in Supplemental Figure S8A (44). As anticipated, there is a strong correlation between the number of differentially-regulated transcripts and the strength of the type 1 functional response as determined by the number of cytokines expressed, although this failed to reach statistical significance (*p* = 0.08; Supplemental Figure S8B). Overall, our data show that polyfunctional T cells are the most transcriptionally active subset of CMV-specific T cells examined (Supplemental Figure S8C). The number of genes that are uniquely and commonly regulated between the functional subsets is displayed in Supplemental Figure S9. We next checked for internal consistency by examining the levels of type 1 cytokine transcriptional and protein expression between the functional subsets (Figure 3). As anticipated, polyfunctional T cells expressed the highest levels of mRNA for each of the three cytokines examined. While there was a nearly identical pattern of IFNγ mRNA and protein expression, the mRNA expression of TNFα lacked the complexity in expression seen at the protein level in populations ***C***–***E***; alternatively, for IL-2, we observed an inverse pattern, where the transcriptional data captures more complex regulation than the protein-level data for populations ***B***–***D***; these latter effects may be due in part to the more intricate post-transcriptional regulation of TNFα and IL-2 mRNA that dictates translation (45).

We next examined whether transcriptional difference in other cytolytic molecule or cytokine expression were also maintained at the protein level following CMV reactivation (Supplemental Figure S10). We found that similar to the transcriptional data, there is a trend toward increased expression of cytolytic molecule Perforin-1 (PRF1) and the degranulation marker CD107a with increasing functionality, and that polyfunctional T cells express significantly more PRF1 and CD107a than IFNγ monofunctional T cells. Interestingly, we also found that polyfunctional T cells express more IL-4 mRNA and protein than their less functional counterparts, although the relative change in expression of this type 2 cytokine is considerably less than the change seen in type 1 cytokines. Overall, this suggests that polyfunctional T cells maintain a type 1 cytokine environment, which may prevent the deleterious effects of a type 2 microenvironment on Th1 CD8+ T cell differentiation (46). Alternatively, evidence suggests that low levels of IL-4 can actually promote Th1 CD8+ T cell responses to infection (47, 48). Alternatively, IL-17 mRNA was not detected in the bifunctional or polyfunctional T cell populations, with variable, low-level (<20 copies/subject) expression in non- and mono-functional T cells; we found no significant difference in IL-17 protein expression between these functional subsets. Collectively, this data suggests that, in addition to improved functional responses, polyfunctional T cells may also possess improved cytolytic function compared to their less functional counterparts.

Finally, we examined whether transcriptional differences in markers of T cell maturation and antigen experience were also maintained at the protein level following re-activation (Supplemental Figure S11). One complicating factor was the presence of non-antigen-specific T cells—specifically naïve T cell populations—in the non-functional (**pop. A**) and to a lesser extent the IFNγ monofunctional T cell population (**pop. B**), which would significantly bias such analysis (Supplemental Figure S3D). As the HLA class 1 genotypes of our healthy donors were unknown, and their PBMCs did not stain positively with commercially-available CMV-specific dextramers, we performed this analysis in CMV-specific T cells following sequential CMV stimulation. Briefly, PBMCs were stimulated with CMV peptide for 24 h, incubated with low-dose IL-2 (10 U/mL) from days 3 through 6, and then re-stimulated with CMV peptide to identify the functional subsets and perform surface staining. This results in a predominantly effector memory phenotype (CCR7^−^/CD45RA^−/int^) for each of the functional subsets (Supplemental Figures S11A,B), which is consistent with our findings in a cohort of kidney transplant recipients and also in line with multiple studies that suggest that CMV-specific T cells predominantly occupy this effector memory phenotype (Supplemental Figure S11C) (49, 50). While the transcriptional data suggests that polyfunctional cells may occupy more of a memory phenotype (CCR7+/–, CD45RA–) compared to less functional subsets, changes in CD45RA expression are not reflected at the protein level. Similarly, the transcriptional data suggests that the polyfunctional cell subset possess a memory precursor effector cell (MPEC) phenotype (CD127+, KLRG1–) compared to monofunctional T cells; while there is a significant increase in CD127 protein expression on polyfunctional T cells compared to monofunctional cells, there is no significant difference in KLRG1 protein expression, consistent with prior findings in CD4+ T cells (18). These observed differences may be due to the short time-course of the experiments, during which changes at the transcriptional have not yet been translated to the receptor level. Whether such changes in CD127 expression correlated with increased sensitivity to IL-7 remains unclear. PD-1 protein expression across the functional subsets closely correlates with the transcriptional data, which may simply reflect the differing levels of activation across these populations. This is further supported by a similar pattern of expression of CD160. Overall, these data support the hypothesis that significant transcriptional and phenotypic differences exist between functional subsets of CMV-specific T cells.

In terms of other γc common chain receptors, we observed a significant increase in IL-2Rα (CD25; *p* = 0.0016) and IL-21R (*p* = 0.014) mRNA expression between monofunctional and polyfunctional T cells (Supplemental Figure S11D). The increased expression of IL-2Rα (CD25) is not unexpected, as CD25 is potently induced upon T cell activation, and the increased expression of IL-2Rα alone is sufficient to increase IL-2 affinity or signaling in the absence of increased IL-2β and IL-2γ expression (51). The increased expression of IL-21 is of particular interest, as IL-21 has been shown to improve antigen-specific memory T cell function, survival and expansion during adoptive T cell expansion when used in combination with IL-7 and IL-15 (52–56). Interestingly, we did not observe any significant increase in the IL-15 receptor, CD215 (IL15Rα). Whether these changes in mRNA expression are also reflected at the protein level demands further investigation.

Evidence suggests that CMV infection and repeated reactivation leads to the inflation of the CD8+ effector memory cell compartment in peripheral blood, marked by the following: (1) loss of CD27, CD28, and CCR7 expression; (2) gain of CD57 and CX3CR1 expression; (3) enhanced cytolytic function with increased granzyme B and perforin expression; and (4) reduced overall proliferative potential (57–59). The increased expression of CX3CR1, which binds fractalkine and has been associated with vascular inflammation (57, 60), has been of particular interest, as CX3CR1^hi^ cells have been shown to have high cytolytic properties but poor proliferative potential while a subpopulation of CX3CR1^int^ cells have been suggested to occupy a more central memory phenotype with increased proliferative potential (57, 58, 61). We observed no significant change in CD27, CD28, and CD57 expression across the functional subsets of CMV-specific CD8+ T cells (Supplemental Figure S11E). While we did observe a trend toward a significant increase in mRNA expression of T cell chemokines CCL3 (MIP-1a; *p* = 0.0588) and CCL4 (MIP-1b; *p* = 0.13) in polyfunctional T cells, we did not observe such a trend in CX3CR1 mRNA expression across the subsets. Again, whether these changes are sustained at the protein level requires further investigation.

Additionally, we sought to determine if the transcriptional profiles obtained from the CMV-specific functional subsets may identify unique cell surface receptors or proteins that might identify polyfunctional T cells and allow for the isolation or enrichment of these cells in a non-destructive manner. We observed significant transcriptional increases in a number of common markers of T cell activation in polyfunctional T cells, including CD25, CD69, TNFSF5 (CD154), TNFSF8 (CD30L), TNFRSF9 (CD137), TNFSF14 (LIGHT), and CRTAM (Supplemental Figure S11F) (62, 63). As the surface expression of CD69 is inhibited by brefeldin A expression, this prevented a direct comparison of CD69 expression with type 1 cytokine expression (63). However, we found that the frequency of CD8+CD69+ cells was generally higher than the frequency of CD8+IFNγ+ cells, indicating a lack of specificity for type 1 cytokine-producing CD8+ T cells, let alone polyfunctional cells (Supplemental Figure S11G). The use of CD107a provided variable results between individuals, lacking specificity for cytokine-producing cells in some individuals and lacking sensitivity in others (Supplemental Figure S11H). Additionally, we did not observe a significant increase in CD137 protein expression in CMV-specific CD8+ T cells at 6 hours (*not shown*), which is consistent with the literature and suggests that a longer incubation (~18 h) may be necessary (62). Furthermore, the study by Litjens et al. suggests that CD137 staining following CMV peptide stimulation identified non-cytokine producing cells similar to CD69 and was not specific for polyfunctional cells. Alternatively, CD154 staining demonstrated a significantly reduced sensitivity for type 1 cytokine-producing CMV-specific T cells, which is consistent with the literature, and while enriching for cytokine-expressing CMV-specific T cells was not specific for polyfunctional T cells (Supplemental Figure S11I) (64). Similarly, the use an antibody against TNFSF8 (CD30L) demonstrated reduced sensitivity, and while its expression is mildly increased in polyfunctional T cells, the difference in expression was not significant between the subsets and would not be amenable to enrichment or non-destructive sorting of polyfunctional cells (Supplemental Figure S11J). Further optimization of these staining techniques, including the duration of stimulation and time of antibody addition (e.g., in culture during stimulation, surface staining after stimulation, etc.) are necessary.

### Exploration of the Transcriptional and Functional Differences Between Polyfunctional and Monofunctional CMV-Specific CD8+ T Cells

**Figure 4:**
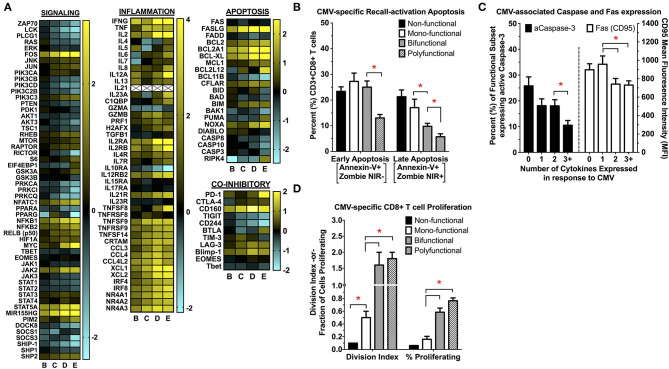
**(A)** Changes in cellular signaling, inflammation, apoptosis/cell survival, and co-inhibitory receptor transcript expression between the functional subsets of CMV-specific CD8+ T cells. First, genes differentially expressed between the functional subsets of CMV-specific CD8+ T cells were identified using DESeq2. This list of differentially expressed genes was then used for further *in silico* analysis, which identified multiple KEGG signaling pathways that were significantly upregulated in polyfunctional vs. mono- or non-functional T cells. This identified pathways involved in TCR- and cytokine-mediated signaling, viral response, inflammation, apoptosis/cell survival, and metabolism. Genes involved in these significantly-regulated pathways were then used to generate the heatmaps shown in (**A**) to highlight specific transcriptional patterns across the subsets (*n* = 5). All values are shown as log_2_ fold-change relative to the non-functional T cell population (**pop A**). The changes in inflammatory signaling seen in polyfunctional T cells is consistent with prior studies examining antigen-specific polyfunctional T cells, identified by the expression of IFNγ alone, without further functional sub-setting to specifically examining polyfunctional cells (65–68). These conserved pathways include tumor necrosis factor receptor (TNFR) signaling, effector/cytokine expression (e.g., GZMB), chemokine signaling (e.g., XCL1/2), and NR4A nuclear receptor family signaling. **(B–D)** Determination of CMV-specific CD8+ T cell re-activation induced apoptosis **(B)**, expression of caspase-3 and FAS (CD95) **(C)**, and proliferation **(D**) following antigen re-challenge (*n* = 5; error bars represent SEM). Apoptosis (early and late), caspase-3, and Fas are expressed as the percentage (%) of the parent population (functional subset), whereas proliferation is expressed as the division index (DI) and the percent (%) parent population. Statistical testing: ^*^p < 0.05. Populations: **(A)** non-functional (IFNγ-/TNFα-/IL-2-); **(B)** IFNγ only/monofunctional (IFNγ+/TNFα-/IL-2-); **(C)** TNFα monofunctional (IFNγ-/TNFα+/IL-2-); **(D)** bifunctional (IFNγ+/TNFα+/IL-2-); and **(E)** polyfunctional (IFNγ+/TNFα+/IL-2+).

To more fully explore the transcriptional differences between CMV-specific CD8+ polyfunctional T cells and the less functional subsets of, we first performed gene-enrichment (GO, GSEA), pathway-specific (KEGG, Ingenuity), and upstream regulator (Ingenuity) analysis between polyfunctional T cells and the less functional subsets (Supplemental Figure S12). We found that a number of pathways were regulated between CMV-specific polyfunctional cells and non- or mono-functional subsets, including those involved in cellular signaling, viral response, proliferation, apoptosis, and metabolism (Figure 4A). Of note, signaling pathways involved in MAPK and JAK-STAT signaling were highly upregulated, as were pathways involved in tumor necrosis factor family signaling. We also found that polyfunctional T cells increased the transcription of substrate transporters and rate-limiting enzymes involved in a number of metabolic pathways, including glycolysis, glutaminolysis and arginine metabolism. Importantly, polyfunctional T cells demonstrated a significant increase in transcripts involved in anti-apoptosis responses, cell cycle progression, and proliferation, indicating that polyfunctional T cells may have distinct survival and proliferative advantages. We then explored whether the transcriptional differences in antigen-induced cell survival and proliferation were maintained at the phenotype level. Following CMV re-encounter, polyfunctional T cells experience significantly less activation-induced apoptosis and capase-3 activation when compared to non-, mono-, and even bi-functional CMV-specific T cells (Figures 4B,C). Moreover, polyfunctional T cells display increased proliferative abilities compared to non- and mono-functional CMV-specific T cells (Figure 4D). This functional dichotomy between polyfunctional and monofunctional T cells is strongly supported in the literature, where only polyfunctional T cells have been associated with protection from CMV (19, 24–28, 33).

**Figure 5:**
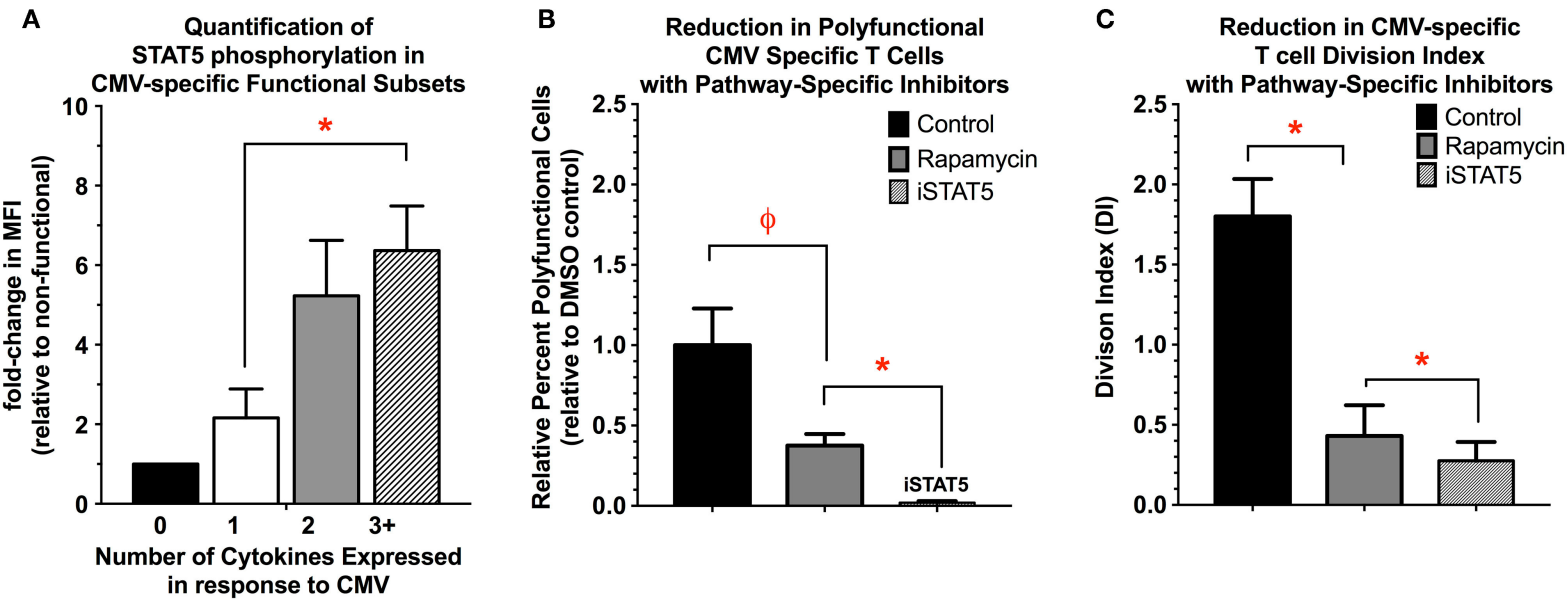
**(A)** Quantification of STAT5 activity in functional subsets of CMV-specific T cells. STAT5 activity was determined by quantifying STAT5 phosphorylation in the functional subsets of CMV-specific T cells, and is expressed as the MFI relative to the non-functional subset (*n* = 4). **(B,C)** Reduction in polyfunctional cell cytokine expression **(B)** and proliferation **(C)** with the use of pathway-specific inhibitors Rapamycin (200 nM) and STAT5 inhibitor (CAS 285986-31-4, 200 μM) (*n* = 4). For cytokine expression, the data are presented as the percent-change in number of polyfunctional T cells relative to DMSO control (0.1% v/v). For proliferation, the data are presented as the division index (DI) for all remaining CMV-reactive T cells (i.e., expressing any type 1 cytokine), as the use of STAT5 inhibitor completely abolished the ability to detect polyfunctional T cells (*n* = 4). Statistical testing: ^ϕ^*p* < 0.005; *p < 0.05.

One of the most significantly regulated pathways between the functional subsets of CMV-specific T cells was the *JAK-STAT* pathway (Supplemental Figures S13A,B). Ingenuity upstream regulator analysis (Qiagen) comparing polyfunctional and monofunctional cells suggests that *STAT5A* may be critical regulator of polyfunctional T cell activity. Evidence suggests that *STAT5* is required for effective CD8+ memory and effector responses and subsequent T cell proliferation (69–75). In polyfunctional cells, there is significant upregulation of *STAT5A/B* as well as several downstream transcriptional targets of *STAT5* activity including *myc, Bcl-2, Bcl-xL, Mcl1, PIM2*, and *Cyclin D1* (72). We next quantified *STAT5A* phosphorylation in CMV-specific functional subsets, finding that *pSTAT5* is significantly increased in polyfunctional T cells relative to mono- and non-functional T cells (Figure 5A). Next, we examined if chemical inhibition of *STAT5* (CAS 285986-31-4, 200 μM) would reduce CMV-specific T cell cytokine expression and proliferation. Rapamycin (200 nM), a potent inhibitor of mTORC1 activity in T cells, was used as a positive control. Inhibition of *STAT5* significantly reduced the number of polyfunctional cells (IFNγ+/TNFα+/IL-2+) compared to solvent or rapamycin treatment. This effect was primarily driven by reduction in IL-2, and less so TNFα expression, as IFNγ expression was largely maintained. Moreover, *STAT5* inhibition also significantly reduced the overall proliferative ability of CMV-specific T cells compared to solvent and rapamycin (Figure 5B). *STAT5* activity therefore appears necessary for polyfunctional cell cytokine production—particularly IL-2—as well as CMV-specific T cell proliferation (Figure 5C).

### Examination of Transcriptional Differences Between Functional Subsets of CMV-Specific T Cells in Immunosuppressed Transplant Recipients

As our prior studies had been performed in healthy subjects, we sought to determine if similar changes in signaling exists between functional subsets of CMV specific T cells in immunosuppressed transplant recipients. However, due to limitations in the number of cells that can safely be obtained from this patient population, this required a single-cell RNAseq (scRNA-seq) approach. Given the strong correlation between transcriptional and protein expression for type 1 cytokines in our healthy subjects, we elected to define functional subsets based on transcription alone. We identified two kidney transplant recipients with similar age and induction immunosuppression and HLA-A^*^0201 genotype, the latter of which allows for dextramer-based detection and isolation of CMV-specific T cells (Supplemental Figure S14). The first subject (248) developed CMV viremia in the post-transplant period, while subject 249 did not. The subjects were matched by age, pre-transplant CMV status (D-/R+), HLA-A^*^0201 status, induction immunosuppression (both received no induction), similar maintenance immunosuppression with similar FK506 target levels, and CMV prophylaxis (both received no CMV prophylaxis). Given both subjects are (D-/R+), we assume pure reactivation in the case subject rather than primary infection. Additionally, the use of patient's that received no induction chemotherapy and no CMV prophylaxis reduces the potential complications that may be associated with lymphodepleting induction regimens and subsequent immune reconstitution and the potential impact that CMV chemoprophylaxis may have on cell-mediated immunity (42, 76). As the case subject developed CMV viremia between the 3- and 6-month visits, samples for both the case and control were selected from the 3-month visit (i.e., prior to development of CMV reactivation in the case subject). Notably, despite these subjects both possessing CMV-specific T cells with an effector memory phenotype, the quality of their T cell-mediated immunity was strikingly different, with subject 248 demonstrating a predominantly non-functional phenotype, and subject 249 with a predominantly polyfunctional response. This dichotomy in functional responses is demonstrated in the PCA visualization (Supplemental Figure S15), where cells from subject 249 express significantly higher levels of cytokines and effector molecules. These differences allowed for estimation of the functional subsets (Supplemental Figure S16). Additionally, when examining a composite of STAT5-related genes (*STAT5A, STAT5B, myc, Bcl2, MIR155HG, PIM2, IL7R*), this composite gene panel is expressed almost exclusively in polyfunctional T cells from subject 249, providing further evidence for role of STAT5 signaling in polyfunctional rather than monofunctional T cells. Finally, the expression of IL-7R, a known upstream regulator of STAT5 activation in T cells, was significantly associated with IFNγ (adjusted *p*-value = 2.30 x 10^−10^) and IL-2 expression (4.24 x 10^−5^). Overall, this suggests that, similar to healthy subjects, polyfunctional T cells in immunosuppressed patients also utilize STAT5 signaling, and therefore engineering of the IL-7/STAT5 axis may allow for the production or preferentially expansion of this unique cell population for therapeutic use against CMV.

## Discussion

### Isolation of High-Quality RNA From Aldehyde-Fixed and Detergent-Permeabilized Immune Cell Populations

Building upon prior efforts, we developed and optimized a cost-effective protocol for the isolation of high-quality RNA from primary human cells following aldehyde-fixation and detergent-based permeabilization, with the successful application of downstream total RNA sequencing. This protocol results in an acceptable loss of RNA quality, quantity, and complexity during cytoplasmic and nuclear straining of intracellular targets. The isolation and subsequent characterization of many cell types of interest require quantification of intracellular targets, including but not limited to cytokines, kinases, and transcription factors. Several prior publications have proposed methods for the isolation of high-quality RNA from fixed and permeabilized samples, including the use of RNase inhibitors, high-salt buffers, zinc-based buffers, as well as more modern, completely reversible fixatives (e.g., DSP, SPDP) (8–16); however, each of these methods has potential drawbacks. Zinc salt-based fixatives, which do not result in crosslinking, have been shown to maintain RNA integrity when coupled with saponin-permeabilization and intracellular staining, and their effect on downstream sequencing protocols remains unclear (10, 11, 77). However, given the lack of RNA crosslinking, there is the potential for significant RNA loss during permeabilization steps. The reversible fixatives DSP and SPDP have also been associated with potential loss not only of RNA mass but also complexity following permeabilization (78). RNase inhibitors, while effective, have varying optimal binding conditions, are often limited to specific RNAses, and may become prohibitively expensive depending on the volumes used for staining, washing, and sorting samples. High-salt buffers, while offering a more economical approach, may significantly interfere with antibody binding in a clone-specific manner, complicating assay optimization (17).

Extracting elements from these prior efforts, our modified protocol has been optimized for the use of aldehyde-based fixatives, for which the majority of antibodies against intracellular targets have been quality-tested; however, this protocol could in theory be used with any of the fixative options listed above. Additionally, by utilizing RNase inhibitors only during low-volume steps (fixation, staining), this protocol reduces overall cost per sample while minimizing the deleterious effects of a high-salt buffer on antibody binding during the intracellular staining steps. This modified protocol results in minimal loss of RNA integrity and quality during cytoplasmic staining of cytokines, while limiting the extent of RNA degradation during nuclear staining of transcription factors. Additionally, this method yielded only a 18% reduction in total RNA yield which is generally tolerable for next-generation sequencing methods.

We have not tested this modified method for use in single cell sequencing experiments. The digestion protocol used for isolating RNA from aldehyde-fixed samples includes an anionic surfactant (SDS), proteinase K, and a temperature-based crosslinking reversal step. This would require a purification step prior to any downstream enzymatic processing such as reverse transcription, which could be accomplished with silica-based magnetic bead isolation. A poly-T isolation approach would lead to increased 3′ bias due to RNA fragmentation that occurs during fixation and permeabilization, thus requiring either a targeted primer amplification or a method that allows for capturing full-length sequence information such as SMART technology or random primers (5). Alternatively, recent studies have shown that cell fixation using precipitant fixatives (e.g., methanol) can be easily reversed by simple rehydration, and yields high quality RNA for downstream single-cell sequencing (79). Unfortunately, alcohol-based fixation and permeabilization is not compatible with many antibody clones and/or fluorophores that are necessary for identification of immune cell subsets of interest. More recently, the RAID protocol was introduced for the combined analysis of intracellular phosphoprotein concentrations and transcriptomics in single cells (80). This protocol utilizes fixation with chemically-reversible fixatives (DSP, SPDP), permeabilization with a detergent supplemented with RNase inhibitor, and cross-linking reversal with DTT. By eliminating protein-RNA crosslinking and the need for SDS and proteinase K reversal, this protocol greatly simplifies downstream processing prior to library generation. However, the use of such mild cross-linkers, which primarily or only cross-link reactive groups in proteins, does increase the risk for loss of RNA mass and complexity during permeabilization steps (78).

Additionally, based on the transcriptional data, we attempted to identify cell surface receptors or other proteins that may uniquely identify polyfunctional T cells and, therefore, allow for the enrichment or isolation of these cells in a non-destructive manner. While several of these techniques proved useful for the enrichment of CMV-specific T cells, none of the molecules tested (CD25, CD69, CD137, CD154, CD30L) was able to differentiate the polyfunctional T cell population with enough resolution to allow for sorting. Additionally, each of these approaches led to either the inclusion of cells not expressing type 1 cytokines (CD25, CD69) or the unacceptable loss of CMV-specific T cells (CD154, CD30L). As we have demonstrated, the use of HLA-specific CMV-loaded multimers, while able to identify CMV-specific T cells in a non-destructive manner, does not provide differentiation of functional attributes such as cytokine polyvalency. Additionally, the use of these single peptide-restricted multimers does not fully characterize the breadth of CMV-specific T cell responses within an individual and require *a priori* knowledge of the donor's HLA genotype. Moreover, activation of CMV-specific T cells with overlapping peptide prior to multimer staining can significantly reduce CD8+ and TCR expression, significantly hampering multimer binding (81). We overcame this limitation by stimulating cells with the multimer in the presence of αCD28 and αCD49d, but this approach again fails to identify the entire breadth of the CMV response as it is limited to a single restricted peptide. Perhaps some combination of the CMV multimers and the above activation markers may provide improved resolution of polyfunctional T cells. Additionally, further optimization of the staining parameters—including duration of stimulation, use of protein transport inhibitors, the timing of antibody addition (e.g., in culture during stimulation vs. surface staining), and use of receptor:ligand blockade—may prove fruitful.

Recently, commercially available cytokine capture kits have been used for the isolation of live monofunctional and polyfunctional *Plasmodium falciparum*-specific CD4+ T cells for subsequent downstream microarray analysis (18, 82). This approach offers a unique advantage over intracellular staining as the isolated cells may be used in downstream propagation or functional studies that require live cells. We have also tested this approach for the isolation of CMV-specific CD8+ T cells which, while feasible, demonstrated a reduced ability to resolve functional subset populations compared to intracellular staining protocols (Supplemental Figure S17). The potential advantages and disadvantages of both the cytokine capture and the intracellular staining approaches are summarized in Supplemental Table S2.

### Transcriptional Profile of CMV-Specific CD8+ T Cell Functional Subsets Obtained Using the Modified Protocol

This study is, to our knowledge, the first to provide the transcriptional profiles for functional subsets of CD8+ T cells, most notably CD8+ polyfunctional cells. Our analysis demonstrated that these functional subsets of CD8+ CMV-specific T cells are molecularly distinct, and that these transcriptional changes were maintained at the cellular level, with polyfunctional T cells demonstrating increased cytolytic molecule expression, reduced activation-induced apoptosis and increased proliferation after antigen re-challenge. *In silico* analysis of the transcriptional data suggested a critical role for *STAT5* transcriptional activity in polyfunctional cell activation, which was confirmed using phosphoprotein staining. Pharmacologic inhibition of *STAT5* was associated with a significant reduction in polyfunctional cell cytokine expression and proliferation, demonstrating the requirement of STAT5 activity not only for proliferation and cell survival, but also cytokine expression. We finally confirmed the association of CMV-specific CD8+ polyfunctionality with STAT5 signaling in immunosuppressed transplant recipients using single cell transcriptomics.

Previous studies have suggested that polyfunctional T cells are correlated with improved target cell killing, pathogen clearance, and clinical outcomes in both pathogen- and malignancy-based models (18–25). However, few prior studies have directly explored the mechanistic differences that drive the function and survival of antigen-specific polyfunctional T cells, as opposed to their less functional counterparts. Burel et al. recently reported on the transcriptional profile of *Plasmodium falciparum*- and *influenza-*specific polyfunctional CD4+ T cells (18), identifying significant differences in anti-infectious and cytokine signaling between polyfunctional and monofunctional cells that was largely conserved between the two pathogens. However, the bulk of the genes involved in the CD4+ polyfunctional signature identified were not differentially regulated in our CD8+ CMV model, suggesting that important differences may exist between CD4+ and CD8+ cells or in the response to different antigens. In human *HIV*- and *influenza*-specific CD8+ T cells, Chiu et al. found that ERK activation was associated with increasing polyfunctionality, and inhibition of ERK activity significant reduced cytokine expression and polyfunctionality (20). Interestingly, evidence suggests that ERK and STAT5 signaling may be closely regulated during T cell activation, either via a common upstream regulator (JAK) or through direct interaction (83, 84).

In this study, we demonstrated that STAT5 activity is required for antigen-induced polyfunctionality and maximal proliferation in healthy subjects, and that a strong association between STAT5 signaling with polyfunctional T cells is maintained in immunosuppressed patients. STAT5 has been shown to play a critical role in antigen-specific effector and memory cell survival and proliferation, although its role in polyfunctional T cell activity has not previously been explored (69–75, 83, 85, 86). STAT5 activity downstream of TCR and/or IL-2 has also been shown to function as a stabilizer of gene regulation initiated following antigen re-exposure (84). Additionally, transfection with constitutively-active STAT5 has been associated with superior anti-tumor activity in mouse models of malignancy, and CAR-T cells engineered toward increased STAT5 activity demonstrate improved proliferation and polyfunctionality while preventing terminal differentiation (71, 72, 74, 75, 86). Several studies have also demonstrated that *ex-vivo* expansion of antigen-specific T cells in the presence of IL-7, an upstream regulator of STAT5 activity, is associated with a STAT5-dependent increase in polyfunctional cells (69–74, 85, 87–89). This is of particular interest given that polyfunctional T cells have increased surface expression of IL-7R, which regulates IL-7 activity. Moreover, this IL-7/STAT5-drive expansion is associated with increased chromatin accessibility in the promoter region of multiple effector genes, suggesting that STAT5 dependence in polyfunctional T cells may be epigenetically imprinted during prior antigen encounters (88).

The importance of IL-7 and STAT5 signaling for CMV immunity in immunosuppressed patients has been suggested previously (90). Moreover, the recent use of JAK/STAT inhibitors for prevention of acute rejection in kidney transplant recipients has been associated with a significantly increased risk of CMV reactivation (91). Bak et al. examined the CMV-specific immunomodulatory effects of the mTORC1 inhibitor sirolimus (rapamycin), finding that not only did therapeutic concentrations (i.e., 10 nM) of sirolimus—which only partially inhibition of mTORC1 activity—improved CMV-specific CD8+ T cell functionality and STAT5 phosphorylation (49). This suggests potential cross-talk between mTORC1 and STAT5 signaling. The mTORC1 complex is necessary for CD8+ T cell effector function; however, persistent mTORC1 activity has been associated with inhibition of cellular processes necessary for the long-term survival of antigen-specific T cells, leads to terminal differentiation and a reduction in IL-2R and IL-7R expression (92–94). Importantly, rapamycin can rescue memory cells from mTORC1 overactivity. As STAT5 activity is primarily dependent upon cytokine receptor signaling, it is possible that unchecked mTORC1 activity may lead to reductions in cytokine signaling through STAT5, and that modulation of mTORC1 activity without complete inhibition is necessary to allow STAT5 activity without compromising effector function and proliferation. This is further supported by evidence that persistent activation of Akt, a primary upstream activator of mTORC1 activity, has been shown to impair LCMV memory cell formation and suppress cytokine expression and STAT5 activity, and that incomplete mTORC1 inhibition can partially rescue this phenomenon (73, 95, 96). Moreover, inhibition of Akt during adoptive T cell expansion has been associated with improved antigen-specific T cell functionality (95–98). This fine-tuning of antigen-specific memory and effector CD8+ T cell function by modulating the balance of activation through the Akt/mTORC1 and JAK/STAT5 pathways offers significant promise for the development new cellular therapeutics.

### Future Directions and Limitations

The results from this study have also generated a number of important new questions. As STAT5 is a downstream target of IL-2 signaling, and IL-2 expression is required for polyfunctionality, this suggests that autocrine IL-2 signaling may be required for polyfunctional cells (99, 100). Second, a number of factors may impact polyfunctional responses in T cells during antigen re-activation, including antigen presentation-specific factors (e.g., co-stimulation/inhibitory receptors, antigen sequence and load, cytokine milieu), TCR-specific factors (e.g., TCR sequence, antigen affinity), and epigenetic imprinting from prior antigen exposures (20, 101–103). Our single cell data, although limited to two subjects, suggests that the antigen sequence is not the sole driving factor, and the pattern of our transcriptional data strongly supports the likelihood that epigenetic changes dictate polyfunctional vs. less functional responses (104–107). Finally, a recent study by Hudson et al. examined long non-coding RNAs (lncRNAs) that were differentially expressed between effector, memory, and naive cells following LCMV Armstrong exposure in mice and yellow fever vaccination in humans (108). Similarly, we found a number of lncRNAs that were differentially expressed between the functional subsets of CMV-specific T cells, suggesting that lncRNAs may be involved in cell fate decisions of polyfunctional T cells. However, the function of the lncRNAs remains unclear.

There are several limitations to the current study. First, the current study is limited to antigen recall in the setting of CMV, a highly immunomodulatory virus associated with terminal differentiation and senescence over the course of life. While our data is largely in line with other studies of anti-viral immunity, it is unclear if these findings will hold for other pathogen classes or in the setting of malignancy. Second, we examined only three type 1 cytokines (IFNγ, TNFα, IL-2) and five functional subsets across three healthy and two immunosuppressed subjects. Given the diversity of CD4+ and CD8+ subpopulations and their associated cytokines and transcription factors, there are multiple distinct polyfunctional cell populations that remain uncharacterized. The recent advances in single cell sequencing should vastly reduce the number of cells required for such studies. Finally, the current study used overlapping peptide stimulation for 6 h in the presence of protein transport inhibitors brefeldin A and monensin. It remains unknown what the effects of antigen presentation, duration and intensity of stimulation, and reduced paracrine/autocrine signaling (due to protein transport inhibitors) play in the transcriptional response of polyfunctional cells.

## Conclusion

In conclusion, this study provides the transcriptional profiles for CMV-specific CD8+ T cell functional subsets, demonstrating that CD8+ polyfunctional T cells are molecularly distinct from their less functional counterparts. Based on prior studies, we developed a modified protocol for the isolation of RNA from aldehyde-fixed, saponin-permeabilized T cells that maintains RNA yield, integrity, and complexity suitable for RNAseq. We identify multiple signaling, survival, and metabolic pathways that are upregulated in CD8+ polyfunctional cells, and that STAT5 signaling is required for polyfunctionality and optimal cell proliferation following antigen-exposure. We then confirmed the association of STAT5 transcriptional activity and polyfunctional in immunosuppressed subjects using single cell sequencing. Overall, the results from this study provide new insights as the mechanisms that drive the generation, function, and survival of polyfunctional T cells. Given the critical importance of polyfunctional cells in anti-viral and anti-tumor immunity, such results could also be extrapolated to the development of vaccines, the treatment of other viral/opportunistic infections, and in the treatment of malignancy.

## Data Availability Statement

Total RNAseq data from bulk CMV-specific CD8+ T cell functional subsets (e.g., monofunctional, polyfunctional), for all three healthy, normal donors is publicly available on GenBank Sequence Read Archive (SRA) under submission **PRJNA613726**. Single-cell RNAseq transcriptional data from CMV-specific CD8+ T cells from the two kidney transplant recipients is publicly available on GenBank Sequence Read Archive (SRA) under submission **PRJNA613687**. For other data included in this report, please direct inquires to the corresponding author.

## Ethics Statement

The studies involving human participants were reviewed and approved by Duke University Hospital IRB. The patients/participants provided their written informed consent to participate in this study.

## Author Contributions

ZH, KW, and DM: conception or design of work, data analysis and interpretation, critical revision, and final approval of the version to be published. ZH and DM: data collection. ZH: drafting the article. All authors contributed to the article and approved the submitted version.

## Conflict of Interest

The authors declare that the research was conducted in the absence of any commercial or financial relationships that could be construed as a potential conflict of interest.

## References

[1] Beliakova-Bethell N Massanella M White C Lada S Du P Vaida F. The effect of cell subset isolation method on gene expression in leukocytes. Cytometry A. (2014) 85:94–104. 10.1002/cyto.a.22352 24115734 PMC3975050

[2] Iglesias-Ussel M Marchionni L Romerio F. Isolation of microarray-quality RNA from primary human cells after intracellular immunostaining and fluorescence-activated cell sorting. J Immunol Methods. (2013) 391:22–30. 10.1016/j.jim.2013.02.003 23434645 PMC3627819

[3] Illumina Evaluating RNA Quality From FFPE Samples. Guidelines for obtaining high-quality RNA sequencing results from degraded RNA with the TruSeq(R) RNA Access Library Preparation Kit.2014.

[4] Schroeder A Mueller O Stocker S Salowsky R Leiber M Gassmann M. The RIN: an RNA integrity number for assigning integrity values to RNA measurements. BMC Mol Biol. (2006) 7:3. 10.1186/1471-2199-7-3 16448564 PMC1413964

[5] Gallego Romero I Pai AA Tung J Gilad Y. RNA-seq: impact of RNA degradation on transcript quantification. BMC Biol. (2014) 12:42. 10.1186/1741-7007-12-42 24885439 PMC4071332

[6] Jones W Greytak S Odeh H Guan P Powers J Bavarva J. Deleterious effects of formalin-fixation and delays to fixation on RNA and miRNA-Seq profiles. Sci Rep. (2019) 9:6980. 10.1038/s41598-019-43282-8 31061401 PMC6502812

[7] Wimmer I Troscher AR Brunner F Rubino SJ Bien CG Weiner HL. Systematic evaluation of RNA quality, microarray data reliability and pathway analysis in fresh, fresh frozen and formalin-fixed paraffin-embedded tissue samples. Sci Rep. (2018) 8:6351. 10.1038/s41598-018-24781-6 29679021 PMC5910432

[8] Brown AL Smith DW. Improved RNA preservation for immunolabeling and laser microdissection. RNA. (2009) 15:2364–74. 10.1261/rna.1733509 19850907 PMC2779672

[9] Esser C Gottlinger C Kremer J Hundeiker C Radbruch A. Isolation of full-size mRNA from ethanol-fixed cells after cellular immunofluorescence staining and fluorescence-activated cell sorting (FACS). Cytometry. (1995) 21:382–6. 10.1002/cyto.990210411 8608737

[10] Jensen UB Owens DM Pedersen S Christensen R. Zinc fixation preserves flow cytometry scatter and fluorescence parameters and allows simultaneous analysis of DNA content and synthesis, and intracellular and surface epitopes. Cytometry A. (2010) 77:798–804. 10.1002/cyto.a.20914 20653019

[11] Lykidis D Van Noorden S Armstrong A Spencer-Dene B Li J Zhuang Z. Novel zinc-based fixative for high quality DNA, RNA and protein analysis. Nucleic Acids Res. (2007) 35:e85. 10.1093/nar/gkm433 17576663 PMC1919503

[12] Nishimoto KP Newkirk D Hou S Fruehauf J Nelson EL. Fluorescence activated cell sorting (FACS) using RNAlater to minimize RNA degradation and perturbation of mRNA expression from cells involved in initial host microbe interactions. J Microbiol Methods. (2007) 70:205–8. 10.1016/j.mimet.2007.03.022 17512621

[13] Pechhold S Stouffer M Walker G Martel R Seligmann B Hang Y. Transcriptional analysis of intracytoplasmically stained, FACS-purified cells by high-throughput, quantitative nuclease protection. Nat Biotechnol. (2009) 27:1038–42. 10.1038/nbt.1579 19838197 PMC4638177

[14] Sandstedt M Jonsson M Asp J Dellgren G Lindahl A Jeppsson A. Intracellular flow cytometry may be combined with good quality and high sensitivity RT-qPCR analysis. Cytometry A. (2015) 87:1079–89. 10.1002/cyto.a.22783 26348124

[15] Hrvatin S Deng F O'Donnell CW Gifford DK Melton DA. MARIS: method for analyzing RNA following intracellular sorting. PLoS ONE. (2014) 9:e89459. 10.1371/journal.pone.0089459 24594682 PMC3940959

[16] Nilsson H Krawczyk KM Johansson ME. High salt buffer improves integrity of RNA after fluorescence-activated cell sorting of intracellular labeled cells. J Biotechnol. (2014) 192(Pt A):62–5. 10.1016/j.jbiotec.2014.09.016 25277986

[17] Kunnath-Velayudhan S Porcelli SA. Isolation of intact RNA from murine CD4(+) T cells after intracellular cytokine staining and fluorescence-activated cell sorting. J Immunol Methods. (2018) 456:77–80. 10.1016/j.jim.2018.02.008 29458078 PMC5878738

[18] Burel JG Apte SH Groves PL McCarthy JS Doolan DL. Polyfunctional and IFN-gamma monofunctional human CD4(+) T cell populations are molecularly distinct. JCI Insight. (2017) 2:e87499. 10.1172/jci.insight.87499 28194431 PMC5291737

[19] Casazza JP Betts MR Price DA Precopio ML Ruff LE Brenchley JM. Acquisition of direct antiviral effector functions by CMV-specific CD4+ T lymphocytes with cellular maturation. J Exp Med. (2006) 203:2865–77. 10.1084/jem.20052246 17158960 PMC2118179

[20] Chiu YL Shan L Huang H Haupt C Bessell C Canaday DH. Sprouty-2 regulates HIV-specific T cell polyfunctionality. J Clin Invest. (2014) 124:198–208. 10.1172/JCI70510 24292711 PMC3871241

[21] Gasser O Bihl F Sanghavi S Rinaldo C Rowe D Hess C. Treatment-dependent loss of polyfunctional CD8+ T-cell responses in HIV-infected kidney transplant recipients is associated with herpesvirus reactivation. Am J Transplant. (2009) 9:794–803. 10.1111/j.1600-6143.2008.02539.x 19298451 PMC2746278

[22] Han Q Bagheri N Bradshaw EM Hafler DA Lauffenburger DA Love JC. Polyfunctional responses by human T cells result from sequential release of cytokines. Proc Natl Acad Sci USA. (2012) 109:1607–12. 10.1073/pnas.1117194109 22160692 PMC3277116

[23] Harari A Dutoit V Cellerai C Bart PA Du Pasquier RA Pantaleo G. Functional signatures of protective antiviral T-cell immunity in human virus infections. Immunol Rev. (2006) 211:236–54. 10.1111/j.0105-2896.2006.00395.x 16824132

[24] Nebbia G Mattes FM Smith C Hainsworth E Kopycinski J Burroughs A. Polyfunctional cytomegalovirus-specific CD4+ and pp65 CD8+ T cells protect against high-level replication after liver transplantation. Am J Transplant. (2008) 8:2590–9. 10.1111/j.1600-6143.2008.02425.x 18853954

[25] Snyder LD Chan C Kwon D Yi JS Martissa JA Copeland CA. Polyfunctional T-cell signatures to predict protection from cytomegalovirus after lung transplantation. Am J Respir Crit Care Med. (2016) 193:78–85. 10.1164/rccm.201504-0733OC 26372850 PMC4731614

[26] Lilleri D Fornara C Chiesa A Caldera D Alessandrino EP Gerna G. Human cytomegalovirus-specific CD4+ and CD8+ T-cell reconstitution in adult allogeneic hematopoietic stem cell transplant recipients and immune control of viral infection. Haematologica. (2008) 93:248–56. 10.3324/haematol.11912 18245650

[27] Lilleri D Gerna G Fornara C Lozza L Maccario R Locatelli F. Prospective simultaneous quantification of human cytomegalovirus-specific CD4+ and CD8+ T-cell reconstitution in young recipients of allogeneic hematopoietic stem cell transplants. Blood. (2006) 108:1406–12. 10.1182/blood-2005-11-012864 16614242

[28] Munoz-Cobo B Solano C Benet I Costa E Remigia MJ de la Camara R. Functional profile of cytomegalovirus (CMV)-specific CD8+ T cells and kinetics of NKG2C+ NK cells associated with the resolution of CMV DNAemia in allogeneic stem cell transplant recipients. J Med Virol. (2012) 84:259–67. 10.1002/jmv.22254 22170546

[29] Camargo JF Wieder E Kimble E Benjamin C Kolonias DS Kwon D. Deep functional immunophenotyping predicts risk of cytomegalovirus reactivation after hematopoietic cell transplantation. Blood. (2018) 133:867–77. 10.1182/blood-2018-10-878918 30573634

[30] Clari MA Munoz-Cobo B Solano C Benet I Costa E Remigia MJ. Performance of the QuantiFERON-cytomegalovirus (CMV) assay for detection and estimation of the magnitude and functionality of the CMV-specific gamma interferon-producing CD8(+) T-cell response in allogeneic stem cell transplant recipients. Clin Vaccine Immunol. (2012) 19:791–6. 10.1128/CVI.05633-11 22379065 PMC3346332

[31] Yong MK Cameron PU Slavin M Morrissey CO Bergin K Spencer A. Identifying cytomegalovirus complications using the quantiferon-CMV assay after allogeneic hematopoietic stem cell transplantation. J Infect Dis. (2017) 215:1684–94. 10.1093/infdis/jix192 28431019

[32] Yong MK Lewin SR Manuel O. Immune monitoring for CMV in transplantation. Curr Infect Dis Rep. (2018) 20:4. 10.1007/s11908-018-0610-4 29542023

[33] Krol L Stuchly J Hubacek P Keslova P Sedlacek P Stary J. Signature profiles of CMV-specific T-cells in patients with CMV reactivation after hematopoietic SCT. Bone Marrow Transplant. (2011) 46:1089–98. 10.1038/bmt.2010.261 21057553

[34] Gimenez E Blanco-Lobo P Munoz-Cobo B Solano C Amat P Perez-Romero P. Role of cytomegalovirus (CMV)-specific polyfunctional CD8+ T-cells and antibodies neutralizing virus epithelial infection in the control of CMV infection in an allogeneic stem-cell transplantation setting. J Gen Virol. (2015) 96:2822–31. 10.1099/vir.0.000203 26025872

[35] Gimenez E Munoz-Cobo B Solano C Amat P de la Camara R Nieto J. Functional patterns of cytomegalovirus (CMV) pp65 and immediate early-1-specific CD8(+) T cells that are associated with protection from and control of CMV DNAemia after allogeneic stem cell transplantation. Transpl Infect Dis. (2015) 17:361–70. 10.1111/tid.12391 25850900

[36] Gerna G Lilleri D Chiesa A Zelini P Furione M Comolli G. Virologic and immunologic monitoring of cytomegalovirus to guide preemptive therapy in solid-organ transplantation. Am J Transplant. (2011) 11:2463–71. 10.1111/j.1600-6143.2011.03636.x 21827612

[37] Gerna G Lilleri D Fornara C Comolli G Lozza L Campana C. Monitoring of human cytomegalovirus-specific CD4 and CD8 T-cell immunity in patients receiving solid organ transplantation. Am J Transplant. (2006) 6:2356–64. 10.1111/j.1600-6143.2006.01488.x 16889599

[38] Gabanti E Bruno F Lilleri D Fornara C Zelini P Cane I. Human cytomegalovirus (HCMV)-specific CD4+ and CD8+ T cells are both required for prevention of HCMV disease in seropositive solid-organ transplant recipients. PLoS ONE. (2014) 9:e106044. 10.1371/journal.pone.0106044 25166270 PMC4148399

[39] Lilleri D Zelini P Fornara C Zavaglio F Rampino T Perez L. Human cytomegalovirus (HCMV)-specific T cell but not neutralizing or IgG binding antibody responses to glycoprotein complexes gB, gHgLgO, and pUL128L correlate with protection against high HCMV viral load reactivation in solid-organ transplant recipients. J Med Virol. (2018) 90:1620–8. 10.1002/jmv.25225 29797330

[40] Lilleri D Gerna G Zelini P Chiesa A Rognoni V Mastronuzzi A. Monitoring of human cytomegalovirus and virus-specific T-cell response in young patients receiving allogeneic hematopoietic stem cell transplantation. PLoS ONE. (2012) 7:e41648. 10.1371/journal.pone.0041648 22848556 PMC3405005

[41] Snyder LD Medinas R Chan C Sparks S Davis WA Palmer SM. Polyfunctional cytomegalovirus-specific immunity in lung transplant recipients receiving valganciclovir prophylaxis. Am J Transplant. (2011) 11:553–60. 10.1111/j.1600-6143.2010.03405.x 21219584 PMC3044779

[42] Razonable RR Humar A. Cytomegalovirus in solid organ transplant recipients-Guidelines of the American society of transplantation infectious diseases community of practice. Clin Transplant. (2019) 33:e13512. 10.1111/ctr.13512 30817026

[43] Wang J Rieder SA Wu J Hayes S Halpin RA de Los Reyes M. Evaluation of ultra-low input RNA sequencing for the study of human T cell transcriptome. Sci Rep. (2019) 9:8445. 10.1038/s41598-019-44902-z 31186477 PMC6559993

[44] Love MI Huber W Anders S. Moderated estimation of fold change and dispersion for RNA-seq data with DESeq2. Genome Biol. (2014) 15:550. 10.1186/s13059-014-0550-8 25516281 PMC4302049

[45] Stamou P Kontoyiannis DL. Posttranscriptional regulation of TNF mRNA: a paradigm of signal-dependent mRNA utilization and its relevance to pathology. Curr Dir Autoimmun. (2010) 11:61–79. 10.1159/000289197 20173387

[46] Fox A Harland KL Kedzierska K Kelso A. Exposure of human CD8(+) T cells to type-2 cytokines impairs division and differentiation and induces limited polarization. Front Immunol. (2018) 9:1141. 10.3389/fimmu.2018.01141 29892290 PMC5985406

[47] Renkema KR Lee JY Lee YJ Hamilton SE Hogquist KA Jameson SC. IL-4 sensitivity shapes the peripheral CD8+ T cell pool and response to infection. J Exp Med. (2016) 213:1319–29. 10.1084/jem.20151359 27298446 PMC4925014

[48] Morris SC Heidorn SM Herbert DR Perkins C Hildeman DA Khodoun MV. Endogenously produced IL-4 nonredundantly stimulates CD8+ T cell proliferation. J Immunol. (2009) 182:1429–38. 10.4049/jimmunol.182.3.1429 19155490 PMC2814185

[49] Bak S Tischer S Dragon A Ravens S Pape L Koenecke C. Selective effects of mTOR inhibitor sirolimus on naive and CMV-specific T cells extending its applicable range beyond immunosuppression. Front Immunol. (2018) 9:2953. 10.3389/fimmu.2018.02953 30619313 PMC6304429

[50] Pourgheysari B Khan N Best D Bruton R Nayak L Moss PA. The cytomegalovirus-specific CD4+ T-cell response expands with age and markedly alters the CD4+ T-cell repertoire. J Virol. (2007) 81:7759–65. 10.1128/JVI.01262-06 17409149 PMC1933343

[51] Lin JX Leonard WJ. The common cytokine receptor gamma chain family of cytokines. Cold Spring Harb Perspect Biol. (2018) 10:a028449. 10.1101/cshperspect.a028449 29038115 PMC6120701

[52] Zoon CK Wan W Graham L Bear HD. Addition of interleukin-21 for expansion of T-cells for adoptive immunotherapy of murine melanoma. Int J Mol Sci. (2015) 16:8744–60. 10.3390/ijms16048744 25903148 PMC4425106

[53] Moretto MM Khan IA. IL-21 is important for induction of KLRG1+ effector CD8 T cells during acute intracellular infection. J Immunol. (2016) 196:375–84. 10.4049/jimmunol.1501258 26597007 PMC4959448

[54] Zoon CK Wan W Graham L Bear HD. Expansion of T cells with interleukin-21 for adoptive immunotherapy of murine mammary carcinoma. Int J Mol Sci. (2017) 18:270. 10.3390/ijms18020270 28146052 PMC5343806

[55] Zeng R Spolski R Finkelstein SE Oh S Kovanen PE Hinrichs CS. Synergy of IL-21 and IL-15 in regulating CD8+ T cell expansion and function. J Exp Med. (2005) 201:139–48. 10.1084/jem.20041057 15630141 PMC2212766

[56] Markley JC Sadelain M. IL-7 and IL-21 are superior to IL-2 and IL-15 in promoting human T cell-mediated rejection of systemic lymphoma in immunodeficient mice. Blood. (2010) 115:3508–19. 10.1182/blood-2009-09-241398 20190192 PMC2867264

[57] Klenerman P. The (gradual) rise of memory inflation. Immunol Rev. (2018) 283:99–112. 10.1111/imr.12653 29664577 PMC5947157

[58] Hertoghs KM Moerland PD van Stijn A Remmerswaal EB Yong SL van de Berg PJ. Molecular profiling of cytomegalovirus-induced human CD8+ T cell differentiation. J Clin Invest. (2010) 120:4077–90. 10.1172/JCI42758 20921622 PMC2964975

[59] Vallejo AN Brandes JC Weyand CM Goronzy JJ. Modulation of CD28 expression: distinct regulatory pathways during activation and replicative senescence. J Immunol. (1999) 162:6572–9. 10352273

[60] Pachnio A Ciaurriz M Begum J Lal N Zuo J Beggs A. Cytomegalovirus infection leads to development of high frequencies of cytotoxic virus-specific CD4+ T cells targeted to vascular endothelium. PLoS Pathog. (2016) 12:e1005832. 10.1371/journal.ppat.1005832 27606804 PMC5015996

[61] Gordon CL Lee LN Swadling L Hutchings C Zinser M Highton AJ. Induction and maintenance of CX3CR1-intermediate peripheral memory CD8(+) T cells by persistent viruses and vaccines. Cell Rep. (2018) 23:768–82. 10.1016/j.celrep.2018.03.074 29669283 PMC5917822

[62] Litjens NH de Wit EA Baan CC Betjes MG. Activation-induced CD137 is a fast assay for identification and multi-parameter flow cytometric analysis of alloreactive T cells. Clin Exp Immunol. (2013) 174:179–91. 10.1111/cei.12152 23750604 PMC3784225

[63] Bacher P Scheffold A. Flow-cytometric analysis of rare antigen-specific T cells. Cytometry A. (2013) 83:692–701. 10.1002/cyto.a.22317 23788442

[64] Han YW Aleyas AG George JA Yoon HA Lee JH Kim BS. Intracellular CD154 expression reflects antigen-specific CD8+ t cells but shows less sensitivity than intracellular cytokine and MHC tetramer staining. J Microbiol Biotechnol. (2007) 17:1955–64. 18167442

[65] Arlehamn CL Seumois G Gerasimova A Huang C Fu Z Yue X. Transcriptional profile of tuberculosis antigen-specific T cells reveals novel multifunctional features. J Immunol. (2014) 193:2931–40. 10.4049/jimmunol.1401151 25092889 PMC4157075

[66] Grifoni A Costa-Ramos P Pham J Tian Y Rosales SL Seumois G. Cutting edge: transcriptional profiling reveals multifunctional and cytotoxic antiviral responses of Zika virus-specific CD8(+) T cells. J Immunol. (2018) 201:3487–91. 10.4049/jimmunol.1801090 30413672 PMC6287102

[67] Grifoni A Tian Y Sette A Weiskopf D. Transcriptomic immune profiles of human flavivirus-specific T-cell responses. Immunology. (2019) 160:3–9. 10.1111/imm.13161 31778581 PMC7160650

[68] Tian Y Babor M Lane J Seumois G Liang S Goonawardhana NDS. Dengue-specific CD8+ T cell subsets display specialized transcriptomic and TCR profiles. J Clin Invest. (2019) 130:1727–41. 10.1172/JCI123726 30882366 PMC6436856

[69] Bitar M Boldt A Freitag MT Gruhn B Kohl U Sack U. Evaluating STAT5 phosphorylation as a mean to assess T cell proliferation. Front Immunol. (2019) 10:722. 10.3389/fimmu.2019.00722 31024554 PMC6460883

[70] Burchill MA Goetz CA Prlic M O'Neil JJ Harmon IR Bensinger SJ. Distinct effects of STAT5 activation on CD4+ and CD8+ T cell homeostasis: development of CD4+CD25+ regulatory T cells versus CD8+ memory T cells. J Immunol. (2003) 171:5853–64. 10.4049/jimmunol.171.11.5853 14634095

[71] Grange M Buferne M Verdeil G Leserman L Schmitt-Verhulst AM Auphan-Anezin N. Activated STAT5 promotes long-lived cytotoxic CD8+ T cells that induce regression of autochthonous melanoma. Cancer Res. (2012) 72:76–87. 10.1158/0008-5472.CAN-11-2187 22065720

[72] Grange M Verdeil G Arnoux F Griffon A Spicuglia S Maurizio J. Active STAT5 regulates T-bet and eomesodermin expression in CD8 T cells and imprints a T-bet-dependent Tc1 program with repressed IL-6/TGF-beta1 signaling. J Immunol. (2013) 191:3712–24. 10.4049/jimmunol.1300319 24006458

[73] Hand TW Cui W Jung YW Sefik E Joshi NS Chandele A. Differential effects of STAT5 and PI3K/AKT signaling on effector and memory CD8 T-cell survival. Proc Natl Acad Sci USA. (2010) 107:16601–6. 10.1073/pnas.1003457107 20823247 PMC2944719

[74] Moriggl R Sexl V Piekorz R Topham D Ihle JN. Stat5 activation is uniquely associated with cytokine signaling in peripheral T cells. Immunity. (1999) 11:225–30. 10.1016/S1074-7613(00)80097-7 10485657

[75] Zimmerman MG Bowen JR McDonald CE Young E Baric RS Pulendran B. STAT5: a target of antagonism by neurotropic flaviviruses. J Virol. (2019) 93:e00665-19. 10.1128/JVI.00665-19 31534033 PMC6854481

[76] Stern A Papanicolaou GA. CMV prevention and treatment in transplantation: what's new in 2019. Curr Infect Dis Rep. (2019) 21:45. 10.1007/s11908-019-0699-0 31732823 PMC8142836

[77] Christensen R Owens DM Thomsen A Pedersen S Jensen UB Zinc fixation for flow cytometry analysis of intracellular and surface epitopes, DNA content, and cell proliferation. Curr Protoc Cytom. (2011) Chapter 7:Unit 7 40. 10.1002/0471142956.cy0740s57 21732310

[78] Xiang CC Mezey E Chen M Key S Ma L Brownstein MJ. Using DSP, a reversible cross-linker, to fix tissue sections for immunostaining, microdissection and expression profiling. Nucleic Acids Res. (2004) 32:e185. 10.1093/nar/gnh185 15604454 PMC545482

[79] Alles J Karaiskos N Praktiknjo SD Grosswendt S Wahle P Ruffault PL. Cell fixation and preservation for droplet-based single-cell transcriptomics. BMC Biol. (2017) 15:44. 10.1186/s12915-017-0383-5 28526029 PMC5438562

[80] Gerlach JP van Buggenum JAG Tanis SEJ Hogeweg M Heuts BMH Muraro MJ. Combined quantification of intracellular (phospho-)proteins and transcriptomics from fixed single cells. Sci Rep. (2019) 9:1469. 10.1038/s41598-018-37977-7 30728416 PMC6365588

[81] Xiao Z Mescher MF Jameson SC. Detuning CD8 T cells: down-regulation of CD8 expression, tetramer binding, and response during CTL activation. J Exp Med. (2007) 204:2667–77. 10.1084/jem.20062376 17954566 PMC2118473

[82] Burel JG Apte SH Doolan DL. Development of a cytokine-secreting-based assay for the identification, sorting and transcriptomic analysis of polyfunctional human T cells. Eur Cytokine Netw. (2015) 26:67–72. 10.1684/ecn.2015.0369 26967764

[83] Pircher TJ Petersen H Gustafsson JA Haldosen LA. Extracellular signal-regulated kinase (ERK) interacts with signal transducer and activator of transcription (STAT) 5a. Mol Endocrinol. (1999) 13:555–65. 10.1210/mend.13.4.0263 10194762

[84] Verdeil G Chaix J Schmitt-Verhulst AM Auphan-Anezin N. Temporal cross-talk between TCR and STAT signals for CD8 T cell effector differentiation. Eur J Immunol. (2006) 36:3090–100. 10.1002/eji.200636347 17111352

[85] Ding ZC Liu C Cao Y Habtetsion T Kuczma M Pi W. IL-7 signaling imparts polyfunctionality and stemness potential to CD4(+) T cells. Oncoimmunology. (2016) 5:e1171445. 10.1080/2162402X.2016.1171445 27471650 PMC4938319

[86] Kagoya Y Tanaka S Guo T Anczurowski M Wang CH Saso K. A novel chimeric antigen receptor containing a JAK-STAT signaling domain mediates superior antitumor effects. Nat Med. (2018) 24:352–9. 10.1038/nm.4478 29400710 PMC5839992

[87] Ding ZC Habtetsion T Cao Y Li T Liu C Kuczma M. Adjuvant IL-7 potentiates adoptive T cell therapy by amplifying and sustaining polyfunctional antitumor CD4+ T cells. Sci Rep. (2017) 7:12168. 10.1038/s41598-017-12488-z 28939858 PMC5610351

[88] Terrazzini N Mantegani P Kern F Fortis C Mondino A Caserta S. Interleukin-7 unveils pathogen-specific T cells by enhancing antigen-recall responses. J Infect Dis. (2018) 217:1997–2007. 10.1093/infdis/jiy096 29506153 PMC5972594

[89] Guler A Lopez Venegas M Adankwah E Mayatepek E Nausch N Jacobsen M. Suppressor of cytokine signalling 3 is crucial for interleukin-7 receptor re-expression after T-cell activation and interleukin-7 dependent proliferation. Eur J Immunol. (2020) 50:234–44. 10.1002/eji.201948302 31621896

[90] Perez-Bercoff L Vudattu NK Byrareddy SN Mattsson J Maeurer M Ljungman P. Reduced IL-7 responsiveness defined by signal transducer and activator of transcription 5 phosphorylation in T cells may be a marker for increased risk of developing cytomegalovirus disease in patients after hematopoietic stem cell transplantation. Biol Blood Marrow Transplant. (2014) 20:128–32. 10.1016/j.bbmt.2013.10.006 24140122

[91] Baan CC Kannegieter NM Felipe CR Tedesco Silva H Jr. Targeting JAK/STAT signaling to prevent rejection after kidney transplantation: a reappraisal. Transplantation. (2016) 100:1833–9. 10.1097/TP.0000000000001226 27163538

[92] Pollizzi KN Patel CH Sun IH Oh MH Waickman AT Wen J. mTORC1 and mTORC2 selectively regulate CD8(+) T cell differentiation. J Clin Invest. (2015) 125:2090–108. 10.1172/JCI77746 25893604 PMC4463194

[93] Delgoffe GM Pollizzi KN Waickman AT Heikamp E Meyers DJ Horton MR. The kinase mTOR regulates the differentiation of helper T cells through the selective activation of signaling by mTORC1 and mTORC2. Nat Immunol. (2011) 12:295–303. 10.1038/ni.2005 21358638 PMC3077821

[94] Powell JD Delgoffe GM. The mammalian target of rapamycin: linking T cell differentiation, function, and metabolism. Immunity. (2010) 33:301–11. 10.1016/j.immuni.2010.09.002 20870173 PMC2962404

[95] Mousset CM Hobo W Ji Y Fredrix H De Giorgi V Allison RD. *Ex vivo* AKT-inhibition facilitates generation of polyfunctional stem cell memory-like CD8(+) T cells for adoptive immunotherapy. Oncoimmunology. (2018) 7:e1488565. 10.1080/2162402X.2018.1488565 30288356 PMC6169586

[96] Urak R Walter M Lim L Wong CW Budde LE Thomas S. *Ex vivo* Akt inhibition promotes the generation of potent CD19CAR T cells for adoptive immunotherapy. J Immunother Cancer. (2017) 5:26. 10.1186/s40425-017-0227-4 28331616 PMC5359873

[97] Zhang Q Ding J Sun S Liu H Lu M Wei X. Akt inhibition at the initial stage of CAR-T preparation enhances the CAR-positive expression rate, memory phenotype and *in vivo* efficacy. Am J Cancer Res. (2019) 9:2379–96. 31815041 PMC6895454

[98] Xu B Yuan L Chen G Li T Zhou J Zhang C. S-15 in combination of Akt inhibitor promotes the expansion of CD45RA(-)CCR7(+) tumor infiltrating lymphocytes with high cytotoxic potential and downregulating PD-1(+)Tim-3(+) cells as well as regulatory T cells. Cancer Cell Int. (2019) 19:322. 10.1186/s12935-019-1043-3 31827396 PMC6889332

[99] Feau S Arens R Togher S Schoenberger SP. Autocrine IL-2 is required for secondary population expansion of CD8(+) memory T cells. Nat Immunol. (2011) 12:908–13. 10.1038/ni.2079 21804558 PMC3388550

[100] Kalia V Sarkar S. Regulation of effector and memory CD8 T cell differentiation by IL-2-a balancing act. Front Immunol. (2018) 9:2987. 10.3389/fimmu.2018.02987 30619342 PMC6306427

[101] Allison KA Sajti E Collier JG Gosselin D Troutman TD Stone EL. Affinity and dose of TCR engagement yield proportional enhancer and gene activity in CD4+ T cells. Elife. (2016) 5:e10134. 10.7554/eLife.10134 27376549 PMC4931909

[102] Conley JM Gallagher MP Berg LJ. T cells and gene regulation: the switching on and turning up of genes after T cell receptor stimulation in CD8 T cells. Front Immunol. (2016) 7:76. 10.3389/fimmu.2016.00076 26973653 PMC4770016

[103] Man K Miasari M Shi W Xin A Henstridge DC Preston S. The transcription factor IRF4 is essential for TCR affinity-mediated metabolic programming and clonal expansion of T cells. Nat Immunol. (2013) 14:1155–65. 10.1038/ni.2710 24056747

[104] Ackermann AM Wang Z Schug J Naji A Kaestner KH. Integration of ATAC-seq and RNA-seq identifies human alpha cell and beta cell signature genes. Mol Metab. (2016) 5:233–44. 10.1016/j.molmet.2016.01.002 26977395 PMC4770267

[105] Hendrickson DG Soifer I Wranik BJ Botstein D Scott McIsaac R. Simultaneous profiling of DNA accessibility and gene expression dynamics with ATAC-seq and RNA-seq. Methods Mol Biol. (2018) 1819:317–33. 10.1007/978-1-4939-8618-7_15 30421411

[106] Jia G Preussner J Chen X Guenther S Yuan X Yekelchyk M. Single cell RNA-seq and ATAC-seq analysis of cardiac progenitor cell transition states and lineage settlement. Nat Commun. (2018) 9:4877. 10.1038/s41467-018-07307-6 30451828 PMC6242939

[107] Ludwig LS Lareau CA Bao EL Nandakumar SK Muus C Ulirsch JC. Transcriptional states and chromatin accessibility underlying human erythropoiesis. Cell Rep. (2019) 27:3228–40.e7. 10.1016/j.celrep.2019.05.046 31189107 PMC6579117

[108] Hudson WH Prokhnevska N Gensheimer J Akondy R McGuire DJ Ahmed R. Expression of novel long noncoding RNAs defines virus-specific effector and memory CD8(+) T cells. Nat Commun. (2019) 10:196. 10.1038/s41467-018-07956-7 30643116 PMC6331603

