# Supplementary material for: Transcriptional Profiling of CD8+ CMV-Specific T Cell Functional Subsets Obtained Using a Modified Method for Isolating High-Quality RNA From Fixed and Permeabilized Cells
Source: Front Immunol. 2020 Sep 2;11:1859. doi: 10.3389/fimmu.2020.01859 (PMC7492549; doi:10.3389/fimmu.2020.01859)
Supplement: Supplementary file 1 [file Data_Sheet_1.pdf]

SUPPLEMENTAL FIGURES

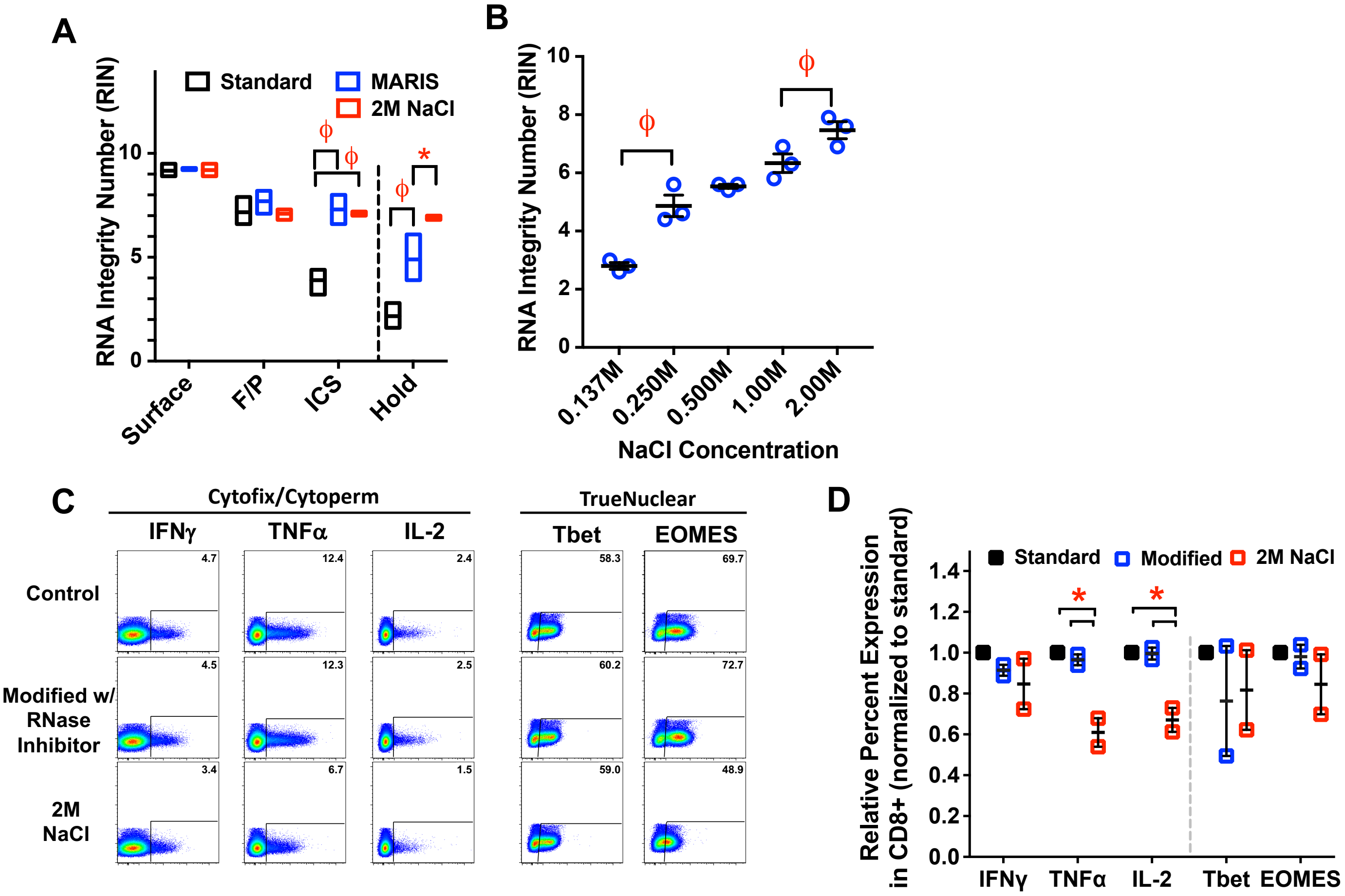

Supplemental Figure S1. (**A**) Comparison of Standard Intracellular Staining (ICS), MARIS, and High Salt (2M NaCl) protocol for the isolation of RNA from fixed and permeabilized samples. Data is represented as the RNA integrity number (RIN) after each of the major steps of the protocol: surface staining, fixation and permeabilization (using PFA 4% and saponin, 0.1%), intracellular staining (using 0.1% saponin), a 2-hour hold to approximate the time spent during transportation to, processing in, and transportation from the sorting facility. Approximately 2 million cells per healthy donor were sampled at each step, and the RNA was isolated using the Ambion FFPE Recover-All kit according to manufacturer's instructions. The integrity of the isolated RNA was then determined on the Agilent 2100 Bioanalyzer. Data were analyzed using paired one-way ANOVA assuming parametric data (n = 3). Considerable degradation of RNA occurring during the intracellular staining process and during the time needed for the process of sorting. While the MARIS protocol, which utilizes a significant quantity of RNAse inhibitor in each buffer at each step following fixation, performs adequately during initial sample processing, this protocol is time sensitive with variable

performance in RIN preservation. Alternatively, a high salt buffer provides significantly more adequate protection from RNase activity. (**B**) The effect of sodium chloride concentration on RNA integrity following processing of samples. Samples from three independent donors were prepared using different concentrations of sodium chloride in the buffers at each step following fixation and permeabilization. All samples underwent CD3-negative magnetic bead isolation (StemCell) prior to RNA isolation (Ambion). Data were analyzed by paired one-way ANOVA assuming parametric data ($n = 3$). As shown in the figure, there is a significant trend ($p < 0.0001$) toward increased RNA degradation as the NaCl concentration is dropped below 2M. There was no added benefit above the 2M concentration (*not shown*) and caused issues with stream charging during cell sorting. (**C-D**) Effect of Intracellular staining buffers containing RNase inhibitor (modified protocol) or 2M NaCl on intracellular target identification. Cryopreserved PBMCs from 2 independent donors were thawed, and approximately $2x10^6$ cells were used for each sample. BD Cytofix/Cytoperm buffer was used during fixation/permeabilization of samples undergoing cytokine staining, while Biolegend TrueNuclear buffer was used during preparation of samples undergoing transcription factor staining. Samples were processed using 2M NaCl in all buffers except during the intracellular staining step. For the control samples, cells underwent intracellular staining in the standard permeabilization buffer provided with these kits. For the RNase inhibitor samples, 1 unit per μL of RNasin plus (Promega) was added to the permeabilization buffer, and for the 2M NaCl samples the buffers were prepared in 2M NaCl solution rather than RNase-free water alone. Samples were then analyzed on a BDFortessa 18-parameter flow cytometer and analyzed using FlowJo software v9.9. Relative expression was gated, analyzed and the resulting population percentage was then expressed relative to the control sample. These ratios were then analyzed using multiple T-tests assuming paired parametric data. While the addition of an RNase inhibitor has no appreciable effect on staining efficiency of any of the cytokines tested, 2M NaCl significantly reduced the staining of TNFα and IL-2, while having variable effects on the staining of transcription factors. Therefore, we elected to utilize a modified protocol, adopting 2M NaCl buffers at all steps except for fixation/permeabilization and intracellular staining ($n = 2$). Statistical significance: (ϕ, $p < 0.005$; ∗, $p < 0.05$).

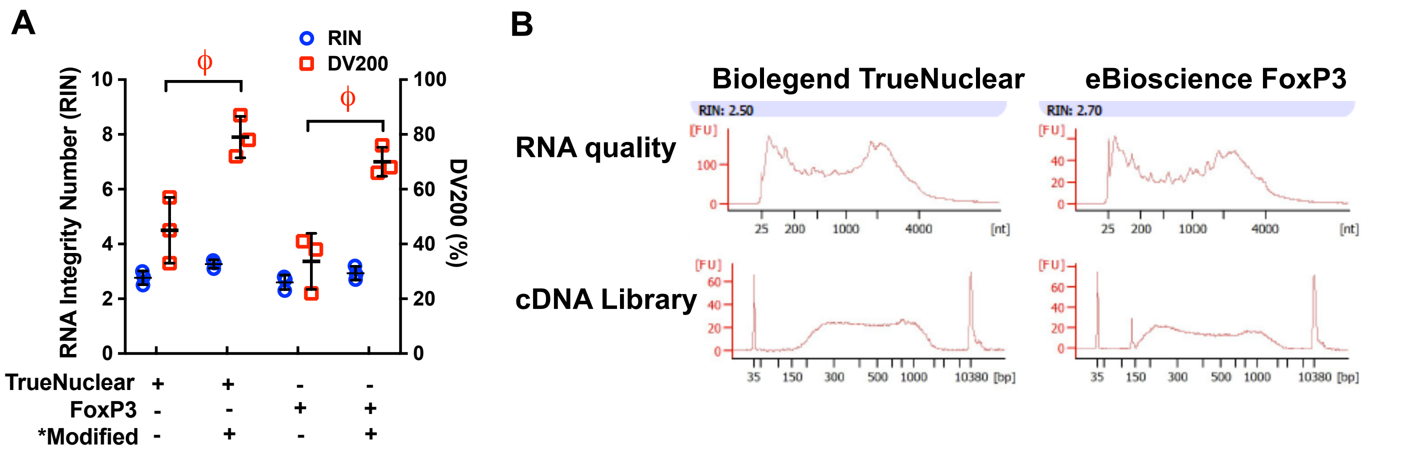

Supplemental Figure S2. (A) Effect of transcription factor fixation and permeabilization buffers on RNA quality. PBMCs from three donors were thawed, rested, surface stained, and subjected to the specified fixation and/or permeabilization protocols. Following fixation, permeabilization, and intracellular staining, cells isolated, and RNA was isolated using either the RNeasy mini kit (live cells) or the Ambion Recoverall FFPE kit (fixed or fixed/permeabilized). RNA quality was determined, and data were analyzed by paired one-way ANOVA (n = 3). The use of stronger permeabilization buffers designed for targeting transcription factors (e.g., eBioscience FoxP3 buffer, Biolegend TrueNuclear buffer) yields considerable RNA degradation. This may be an unavoidable consequence of the conditions necessary to effectively permeabilize the nuclear membrane, leading to increased RNA fragmentation. However, the modified protocol is able to maintain the DV200, an alternative measure of RNA quality used in samples with low RIN values, at much higher values than any commercial protocol[1]. Recent evidence suggests that in RNA samples with significant fragmentation and hence low RIN scores, highly reproducible and reliable RNAseq data may be obtained from samples with DV200 scores greater than 60-70%, while those with lower DV200 scores are generally unreliable[1]. Statistical significance: (ϕ, $p < 0.005$; $*$, $p < 0.05$). (B) Representative RNA electropherograms and cDNA libraries generated from these degraded samples. The libraries generated from these more significantly degraded RNA samples are predictably broad with respect to insert size, which may also negatively impact sequencing (**Supplemental Figure S2B**)[2]. The use of RNA, cDNA, or library size selection techniques may therefore be necessary to generate libraries with more homogenous lengths as dictated by the sequencing technology. Overall, the yield of RNA from samples treated with nuclear permeabilization reagents was not significantly different than that of more mild agents.

This arose despite the use of bead-based size selection during library preparation. Total RNAseq data from bulk CMV-specific CD8+ T cell functional subsets (e.g., monofunctional, polyfunctional), for all three healthy, normal donors is publicly available on GenBank Sequence Read Archive (SRA) under submission **PRJNA613726**. Single-cell RNAseq transcriptional data from CMV-specific CD8+ T cells from the two kidney transplant recipients is publicly available on GenBank Sequence Read Archive (SRA) under submission **PRJNA613687**. For other data included in this report, please direct inquires to the corresponding author.

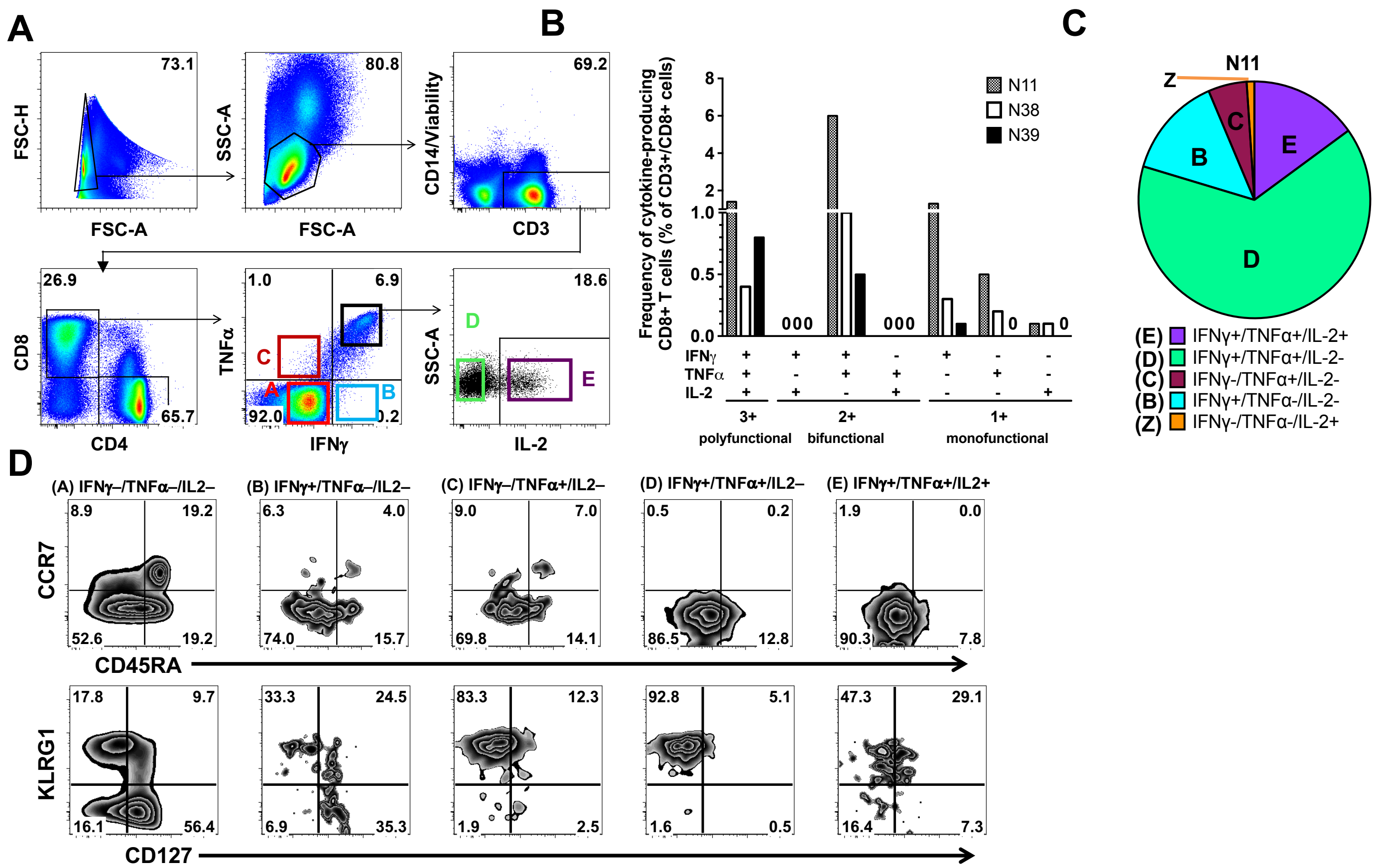

Supplemental Figure S3. (A) Sorting strategy for identification of non-functional, monofunctional, bifunctional, and polyfunctional CMV-specific CD8+ T cells. PBMCs are thawed, rested, and stimulated with overlapping CMV peptide as described for 6 hours in the presence of BFA and monensin. Cells were stained for viability and cell surface markers, then fixed and permeabilized (BD Cytofix/CytoPerm). Intracellular staining was then performed for cytokines or other targets and the cells examined on BDFortessa flow cytometer and analyzed using FlowJo v9.9. Aggregate cells are first excluded, and lymphocytes are then identified by scatter characteristics. Viable CD3+ T cells are then identified and CD4+ and CD8+ lineage subsets are identified. CD8+ T cells are then analyzed for IFNγ and TNFα cytokine production, and IFNγ+/TNFα+ cells are then examined for IL-2 expression. Five populations are identified (A – IFNγ-/TNFα-/IL-2-; B - A – IFNγ+/TNFα-/IL-2-; C - A – IFNγ-/TNFα+/IL-2-; D - A – IFNγ+/TNFα+/IL-2-; E - A – IFNγ-/TNFα-/IL-2-). (B-C) Polyfunctional analysis of CMV-specific CD8+ CMV cells from three independent donors as performed in SPICE. (D) Maturation/differentiation status (CCR7, CD45RA) and markers of antigen experience (CD127, KLRG1) of each functional subset of CMV-specific CD8+ T cells. Populations: (**A**) non-functional (IFNγ-/TNFα-/IL-2-); (**B**) IFNγ only/monofunctional (IFNγ+/TNFα-/IL-2-); (**C**) TNFα

monofunctional (IFNγ-/TNFα+/IL-2-); (**D**) bifunctional (IFNγ+/TNFα+/IL-2-); and (**E**) polyfunctional (IFNγ+/TNFα+/IL-2+).

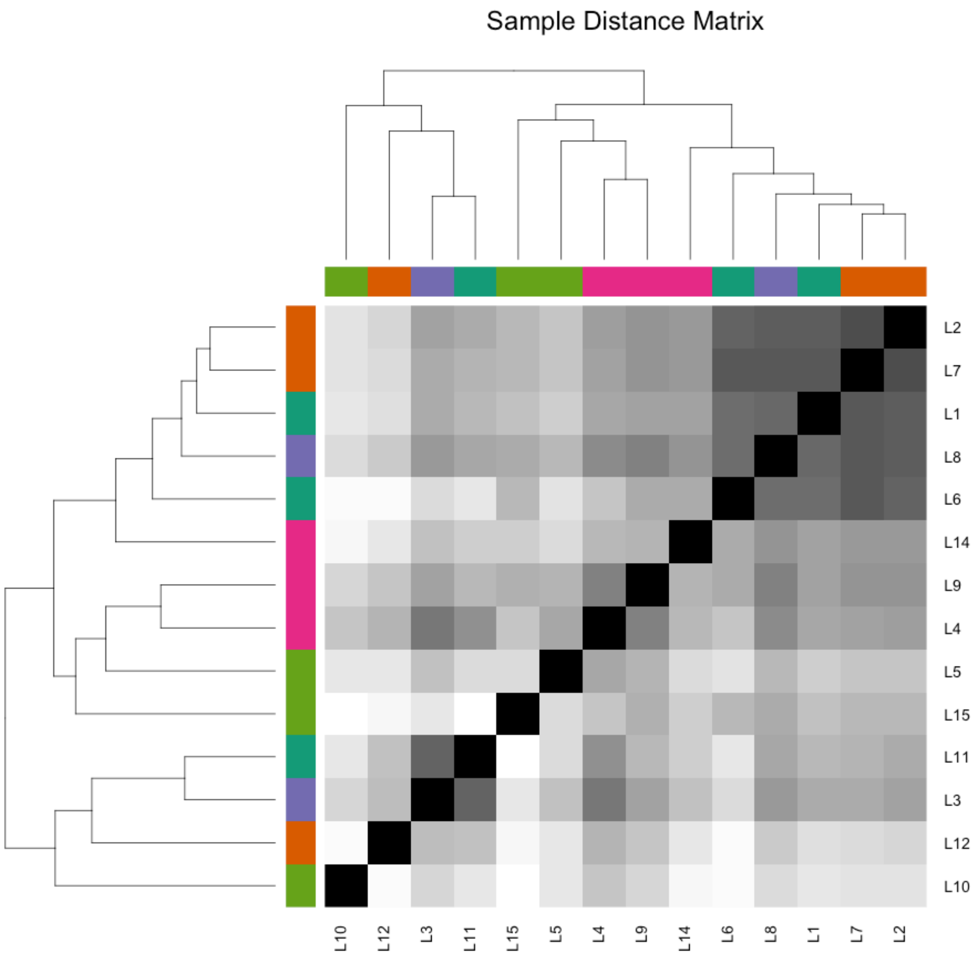

A – L1, L6, L11
B – L2, L7, L12
C – L3, L8
D – L4, L9, L14
E – L5, L10, L15

Donor N11 – L1-L5
Donor N38 – L6-L10
Donor N39 – L11-L15

Supplemental Figure S4. Heatmap of sample-to-sample distances for the 14 individual RNA libraries. The key for the functional subsets and donors is provided on the right. Populations: (**A**) non-functional (IFNγ-/TNFα-/IL-2-); (**B**) IFNγ only/monofunctional (IFNγ+/TNFα-/IL-2-); (**C**) TNFα monofunctional (IFNγ-/TNFα+/IL-2-); (**D**) bifunctional (IFNγ+/TNFα+/IL-2-); and (**E**) polyfunctional (IFNγ+/TNFα+/IL-2+).

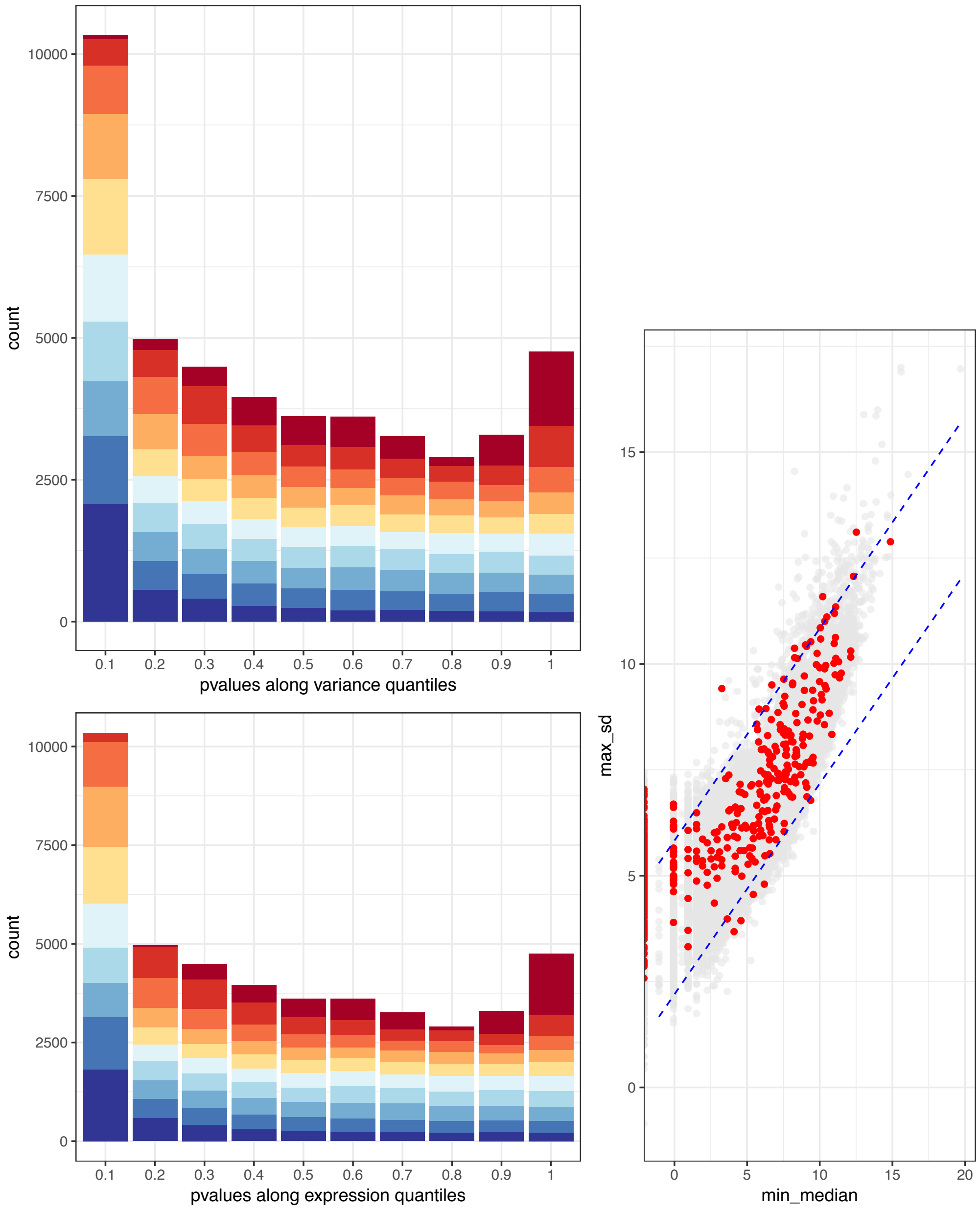

Supplemental Figure S5. Distribution of p-values along variance and expression quartiles (comparison: polyfunctional (***pop. E***) versus IFNγ monofunctional (***pop. B***).

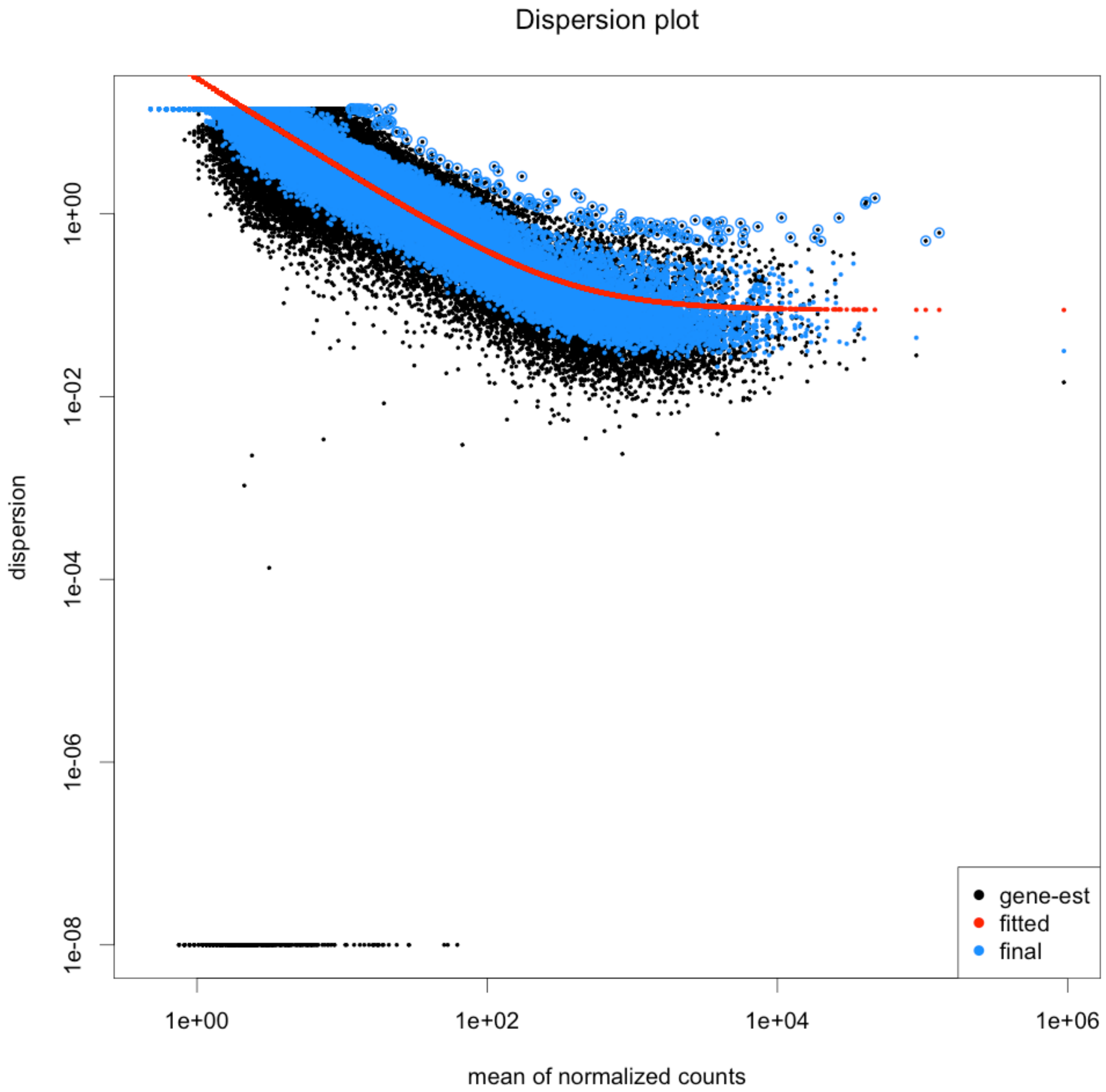

Supplemental Figure S6. Dispersion plot (comparions: polyfunctional (***pop. E***) versus IFNγ monofunctional (***pop. B***).

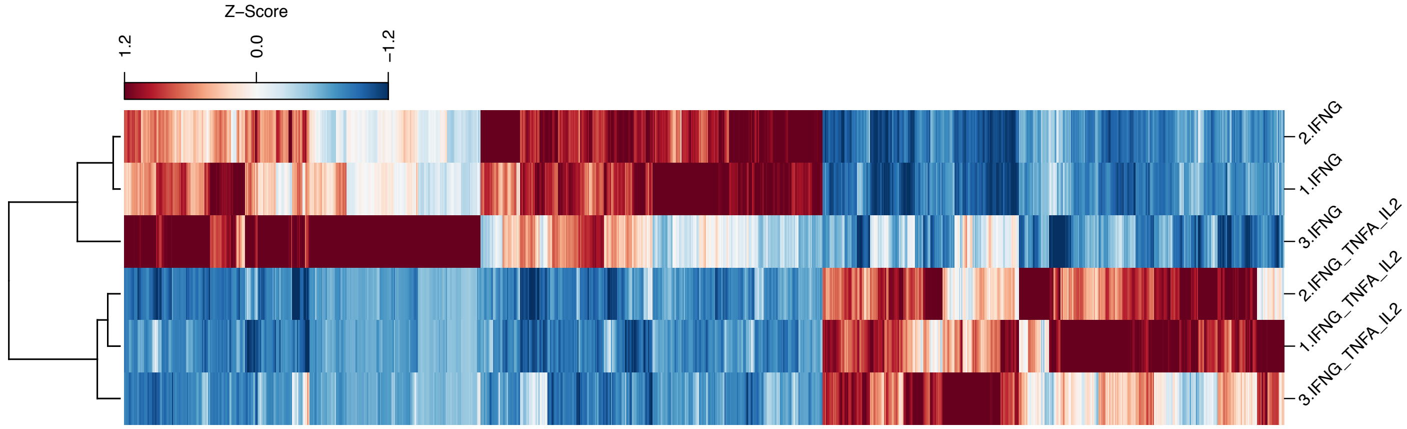

Supplemental Figure S7. Hierarchical clustering analysis (comparison: polyfunctional (***pop. E***) versus IFNγ monofunctional (***pop. B***).

A

Coding	A	B	C	D
A				
B	0 / 0			
C	0 / 0	0 / 0		
D	372 / 29	10 / 6	0 / 0	
E	897 / 545	369 / 360	92 / 104	27 / 70

lncRNA	A	B	C	D
A				
B	0 / 0			
C	0 / 0	0 / 0		
D	52 / 6	2 / 0	0 / 0	
E	78 / 246	33 / 215	6 / 97	10 / 89

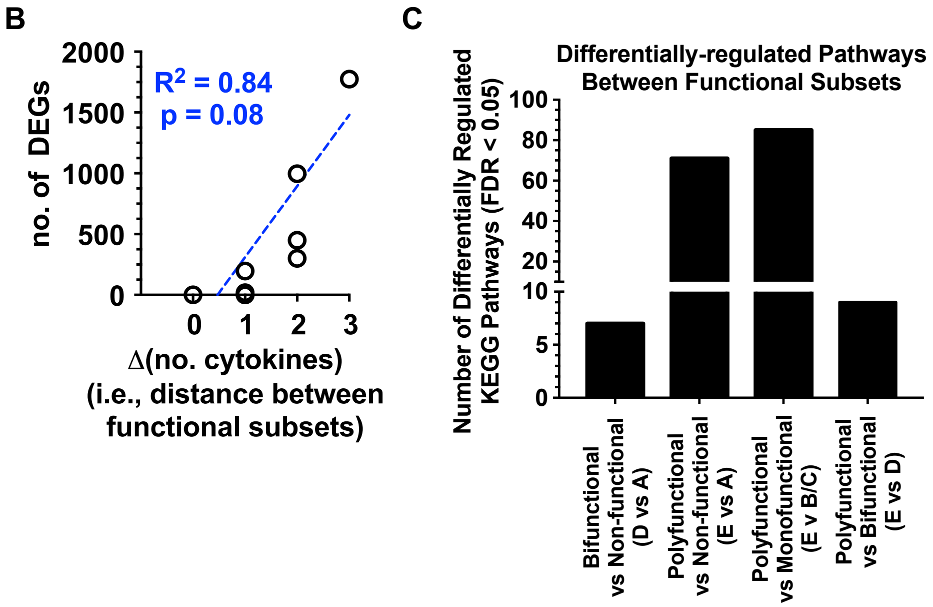

Supplemental Figure S8. (**A**) The number of coding and long non-coding (lnc) RNA transcripts that are significantly up- (**red, top**) or down- (**blue, bottom**) regulated between functional subsets of CMV-specific T cells. Differentially regulated genes were identified using DESeq2. A Venn diagram is provided in Supplemental Figure 9 that demonstrates the overlap of differentially expressed genes between these subsets. (**B**) Linear regression analysis to determine the distance between subsets (i.e., difference in number of cytokines expressed) was correlated with the number of differentially expressed genes. (**C**) Number of differentially-regulated KEGG pathways between functional subsets of CMV-specific CD8+ T cells (FDR < 0.10). Populations: (**A**) non-functional (IFNγ-/TNFα-/IL-2-); (**B**) IFNγ only/monofunctional (IFNγ+/TNFα-/IL-2-); (**C**) TNFα monofunctional (IFNγ-/TNFα+/IL-2-); (**D**) bifunctional (IFNγ+/TNFα+/IL-2-); and (**E**) polyfunctional (IFNγ+/TNFα+/IL-2+)

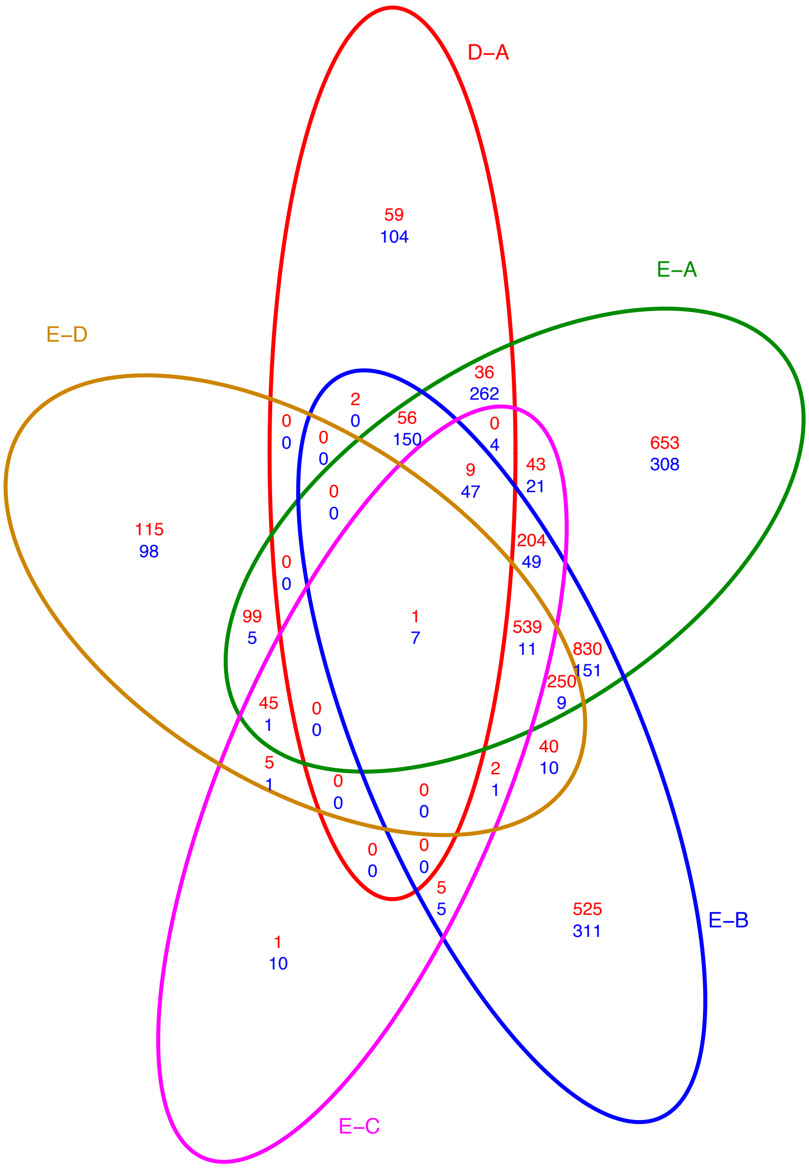

Supplemental Figure S9. Venn diagram for differentially expressed transcripts (both coding and lncRNA). Note again that the number of unique differentially expressed genes between functional subsets is inversely related to the functional distance between the two populations (i.e., EvA = difference of 3 cytokines; EvB and EvC = difference of 2 cytokines; EvD = difference of 1 cytokine). Populations: (**A**) non-functional (IFNγ-/TNFα-/IL-2-); (**B**) IFNγ only/monofunctional (IFNγ+/TNFα-/IL-2-); (**C**) TNFα monofunctional (IFNγ-/TNFα+/IL-2-); (**D**) bifunctional (IFNγ+/TNFα+/IL-2-); and (**E**) polyfunctional (IFNγ+/TNFα+/IL-2+).

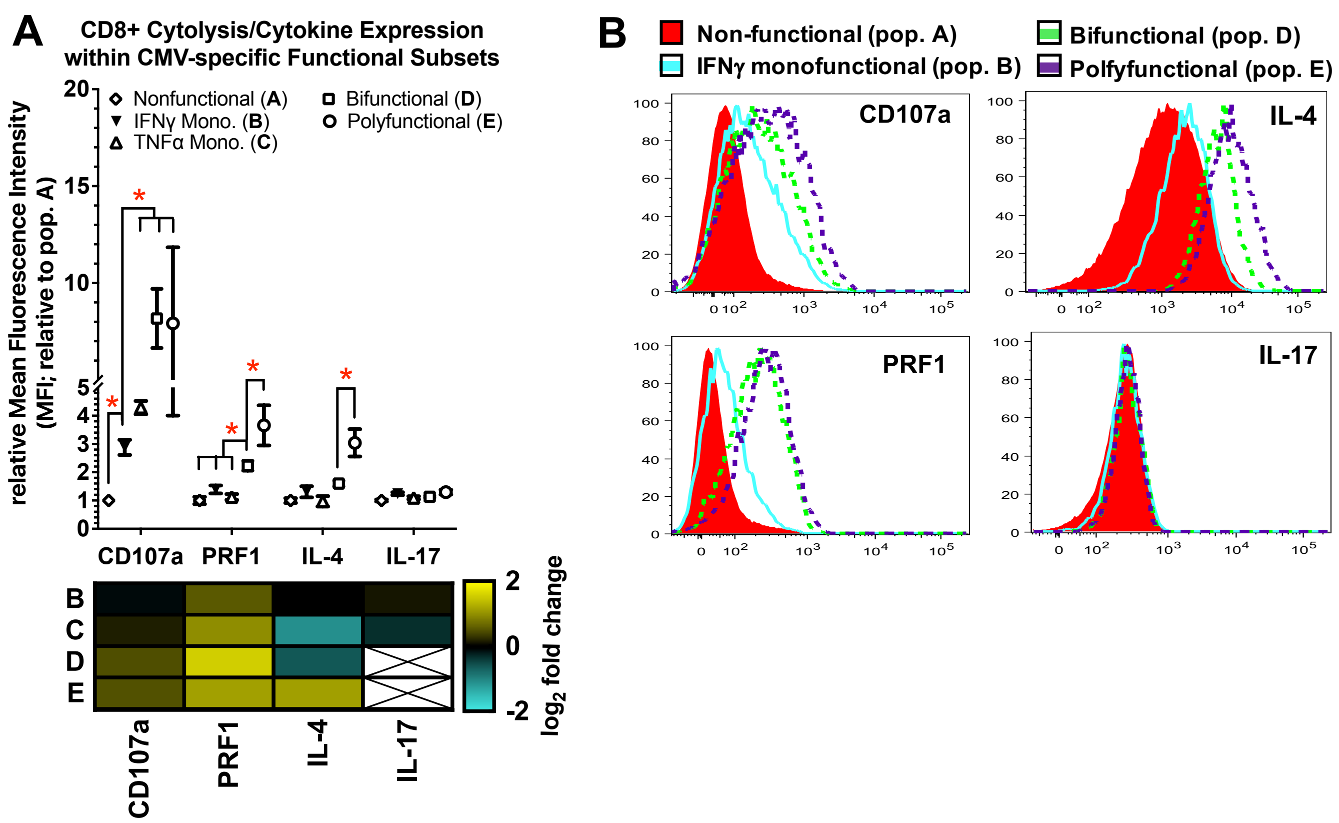

Supplemental Figure S10. Correlation of transcriptional and cytokine/cytolysis molecule expression within functional subsets of CMV-specific T cells. (**A**) A comparison of intracellular protein and transcriptional expression and of degranulation marker CD107a, cytolytic molecule Perforin-1 (PRF1), type 2 cytokine IL-4, and type 17 cytokine IL-17 within the functional subsets of CMV-specific T cells. For protein expression, data are expressed as the change in mean fluorescence intensity relative to nonfunctional cells (***pop. A***). For the transcriptional data, the data is presented in a heatmap and expressed as the fold-change ($\log_2$) relative to the non-functional cell population (***pop. A***). (**B**) Representative overlapping histograms for the individual markers of interest within the functional subsets of CMV-specific T cells. Statistical significance: (ϕ, $p < 0.005$; $*$, $p < 0.05$). *Populations*: (**A**) non-functional (IFNγ-/TNFα-/IL-2-); (**B**) IFNγ only monofunctional (IFNγ+/TNFα-/IL-2-); (**C**) TNFα monofunctional (IFNγ-/TNFα+/IL-2-); (**D**) bifunctional (IFNγ+/TNFα+/IL-2-); and (**E**) polyfunctional (IFNγ+/TNFα+/IL-2+)

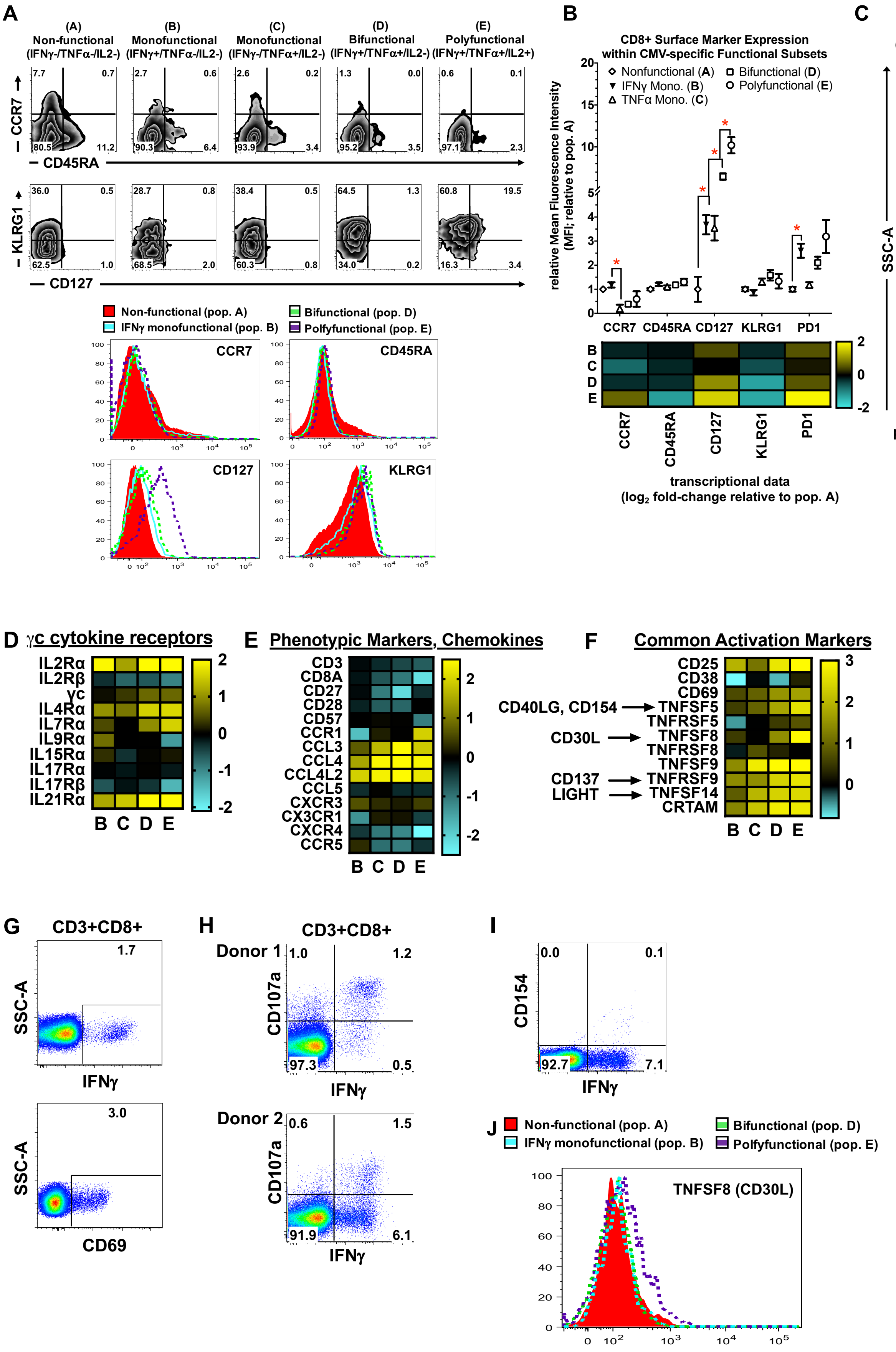

A
(A) Non-functional (IFNγ-/TNFα-/IL2-)
(B) Monofunctional (IFNγ+/TNFα-/IL2-)
(C) Monofunctional (IFNγ-/TNFα+/IL2-)
(D) Bifunctional (IFNγ+/TNFα+/IL2-)
(E) Polyfunctional (IFNγ+/TNFα+/IL2+)
CCR7
CD45RA
KLRG1
CD127
Non-functional (pop. A)
IFNγ monofunctional (pop. B)
Bifunctional (pop. D)
Polyfunctional (pop. E)
B
CD8+ Surface Marker Expression within CMV-specific Functional Subsets
Nonfunctional (A)
IFNγ Mono. (B)
TNFα Mono. (C)
Bifunctional (D)
Polyfunctional (E)
relative Mean Fluorescence Intensity (MFI; relative to pop. A)
CCR7 CD45RA CD127 KLRG1 PD1
transcriptional data (log2 fold-change relative to pop. A)
C
CD3+CD8+
pp65-A0201+
SSC-A
pp65-A0201 dextramer
CCR7
CD45RA
D
γc cytokine receptors
IL2Rα IL2Rβ γc IL4Rα IL7Rα IL9Rα IL15Rα IL17Rα IL17Rβ IL21Rα
B C D E
E
Phenotypic Markers, Chemokines
CD3 CD8A CD27 CD28 CD57 CCR1 CCL3 CCL4 CCL4L2 CCL5 CXCR3 CX3CR1 CXCR4 CCR5
B C D E
F
Common Activation Markers
CD25 CD38 CD69 TNFSF5 TNFRSF5 TNFSF8 TNFRSF8 TNFSF9 TNFRSF9 TNFSF14 CRTAM
CD40LG, CD154
CD30L
CD137
LIGHT
B C D E
G
CD3+CD8+
SSC-A
IFNγ
CD69
H
CD3+CD8+
Donor 1
Donor 2
CD107a
IFNγ
I
CD154
IFNγ
J
Non-functional (pop. A)
IFNγ monofunctional (pop. B)
Bifunctional (pop. D)
Polyfunctional (pop. E)
TNFSF8 (CD30L)

Supplemental Figure S11. (**A-B**) Correlation of transcriptional and protein expression of maturation and antigen experience markers on CMV-specific CD8+ T cell functional subsets. For these experiments, which were performed with the survival and proliferation studies show in Figure 4D, PBMC were first labeled with proliferation dye, stimulated with CMV peptide for 24 hours, and incubated for and additional 5 days with IL-2 (10 U/mL) added on day 3. On Day 6, the cells were re-stimulated with CMV peptide and stained for intracellular cytokines and surface markers as described. This had the effect of reducing naïve T cell contamination seen in the non-functional and to a lesser extent the IFNγ monofunctional T cell populations seen in **Supplemental Figure S3**, leaving a predominantly effector memory population for each of the functional subsets of CMV-specific T cells. Representative maturational and antigen-experience plots for the functional subsets and corresponding overlapping histograms are shown in **Supplemental Figure S11A**. In **S11B**, protein expression data are expressed as the change in mean fluorescence intensity relative to nonfunctional cells (***pop. A***). For the transcriptional data, the data is presented in a heatmap and expressed as the fold-change (log_2) relative to the non-functional cell population (***pop. A***). (**C**) Maturation analysis (CCR7/CD45RA) within pp65-*A0201 specific CD8+ T cells, confirming that at baseline, CMV-specific T cells occupy a predominantly effector phenotype ($CCR7^{-}/CD45RA^{-/int}$). (**D-F**) Transcriptional changes in common chain (γc) cytokine receptor (**D**), phenotypic markers and chemokine (**E**), and markers of activation across the functional subsets of CMV-specific T cells (**F**). (**G**) Comparison of CD69 and IFNγ staining in CD3+CD8+ T cells following stimulation of overlapping CMV peptides, demonstrating that CD69 staining identifies cells that do not express type 1 cytokines. (**H**) Comparison of CD107a and IFNγ staining in CD3+CD8+ T cells following stimulation of overlapping CMV peptides, demonstrating that CD107a provides heterogenous identification of CMV-specific T cells expressing type 1 cytokines, lacking specificity in some donors (Donor 1) and sensitivity in others (Donor 2). (**I**) Comparison of CD154 and IFNγ staining in CD3+CD8+ T cells following stimulation of overlapping CMV peptides, demonstrating a significantly reduced sensitivity for CMV-specific T cells. (**J**) Demonstration that TNFSF8 staining does not reliably resolve polyfunctional T cells from their less functional counterparts. Populations: (**A**) non-functional (IFNγ-/TNFα-/IL-2-); (**B**) IFNγ only/monofunctional (IFNγ+/TNFα-/IL-2-); (**C**) TNFα monofunctional (IFNγ-/TNFα+/IL-2-); (**D**) bifunctional (IFNγ+/TNFα+/IL-2-); and (**E**) polyfunctional (IFNγ+/TNFα+/IL-2+).

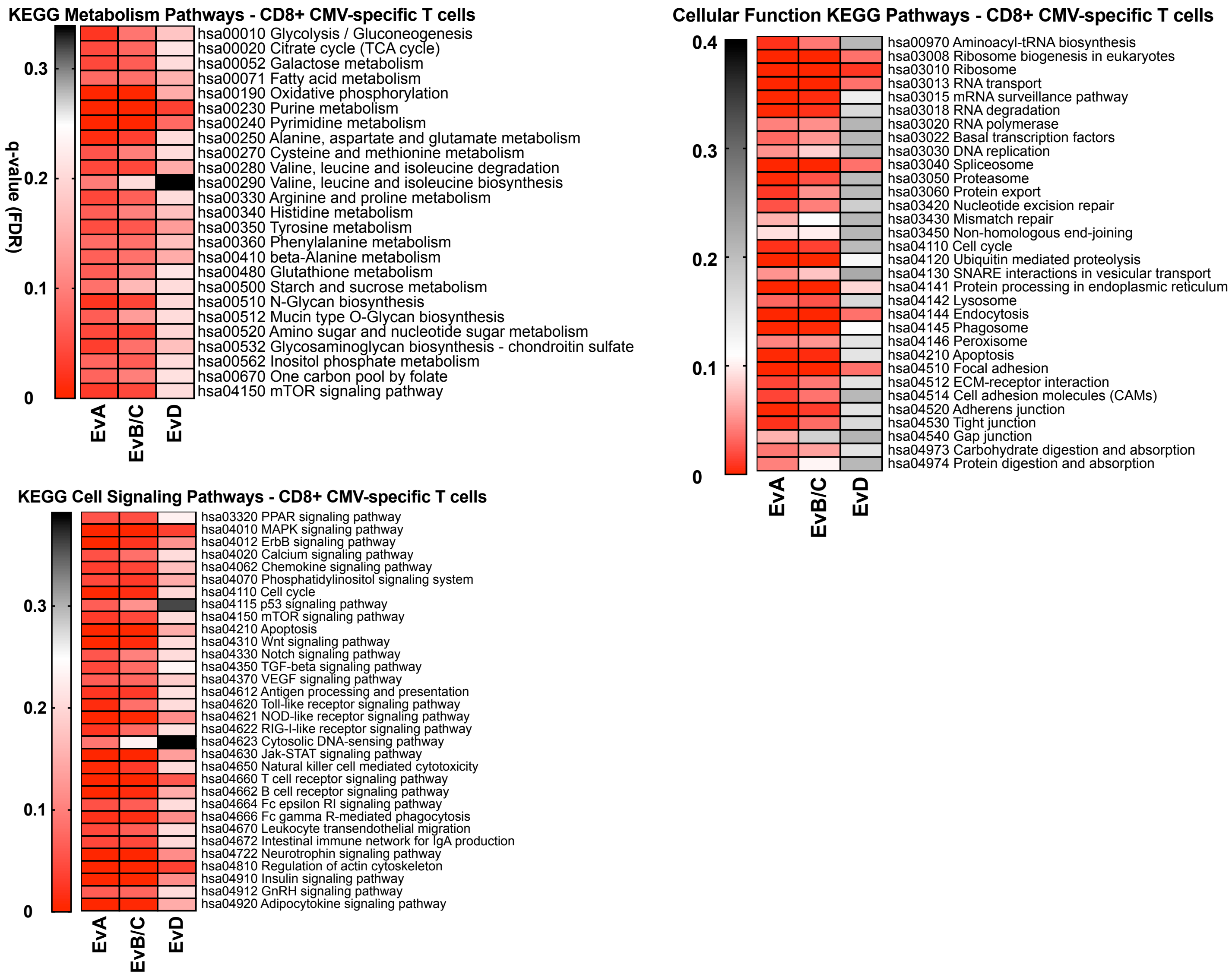

Supplemental Figure S12. KEGG metabolism, cell signaling, and cellular function analysis for CMV-specific polyfunctional CD8+ T cells. Populations: (**A**) non-functional (IFNγ-/TNFα-/IL-2-); (**B**) IFNγ only/monofunctional (IFNγ+/TNFα-/IL-2-); (**C**) TNFα monofunctional (IFNγ-/TNFα+/IL-2-); (**D**) bifunctional (IFNγ+/TNFα+/IL-2-); and (**E**) polyfunctional (IFNγ+/TNFα+/IL-2+).

A

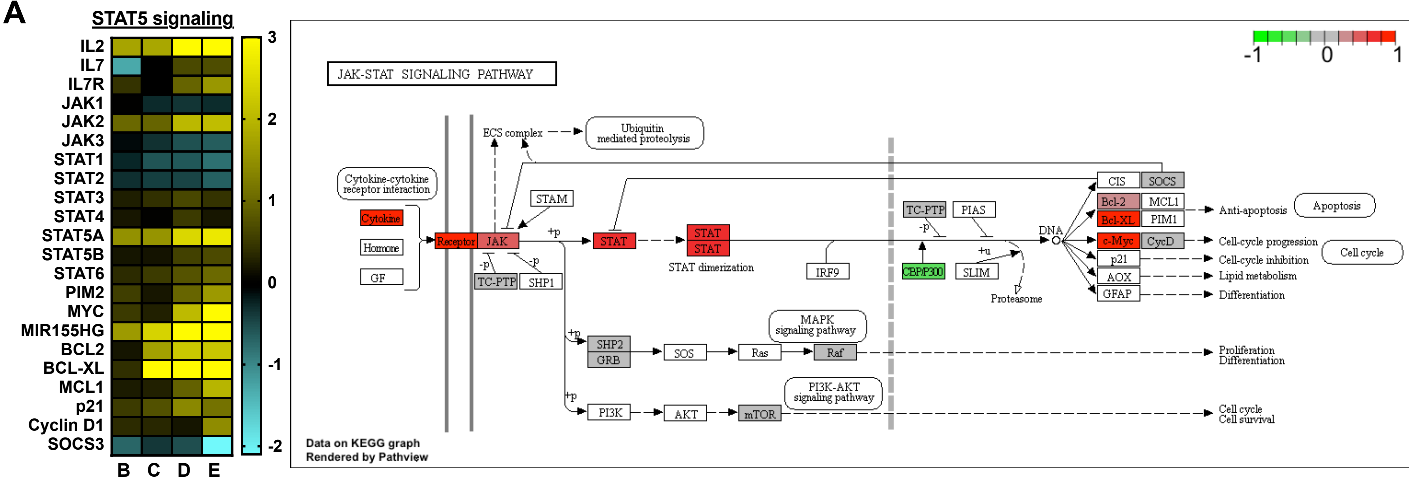

B

Ingenuity Pathway Analysis (IPA) *Upstream Transcriptional Regulators*			
**Rank**	**Upstream Regulator**	**Z-score**	**P value**
1	SATB1	2.226	2.01x10^{-08}
2	ATF4		1.44x10^{-04}
6	MYC	2.335	2.82x10^{-04}
4	REL		9.23x10^{-04}
**5**	**STAT5a/b**	**2.449**	**2.11x10^{-03}**
6	IRF9	2.335	2.82x10^{-04}
7	JUN	1.633	6.3310^{-03}
8	NFkB1	3.504	1.2310^{-03}
9	NFATC1		1.2710^{-03}
10	DRAP1		1.5910^{-02}

Supplemental Figure S13. (**A**) Transcriptional data and graphical representation of STAT5 signaling pathway generated in Pathview. (**B**) Upstream transcriptional regulators identified through Ingenuity Pathway Analysis (IPA; Qiagen). Populations: (**A**) non-functional (IFNγ-/TNFα-/IL-2-); (**B**) IFNγ only/monofunctional (IFNγ+/TNFα-/IL-2-); (**C**) TNFα monofunctional (IFNγ-/TNFα+/IL-2-); (**D**) bifunctional (IFNγ+/TNFα+/IL-2-); and (**E**) polyfunctional (IFNγ+/TNFα+/IL-2+).

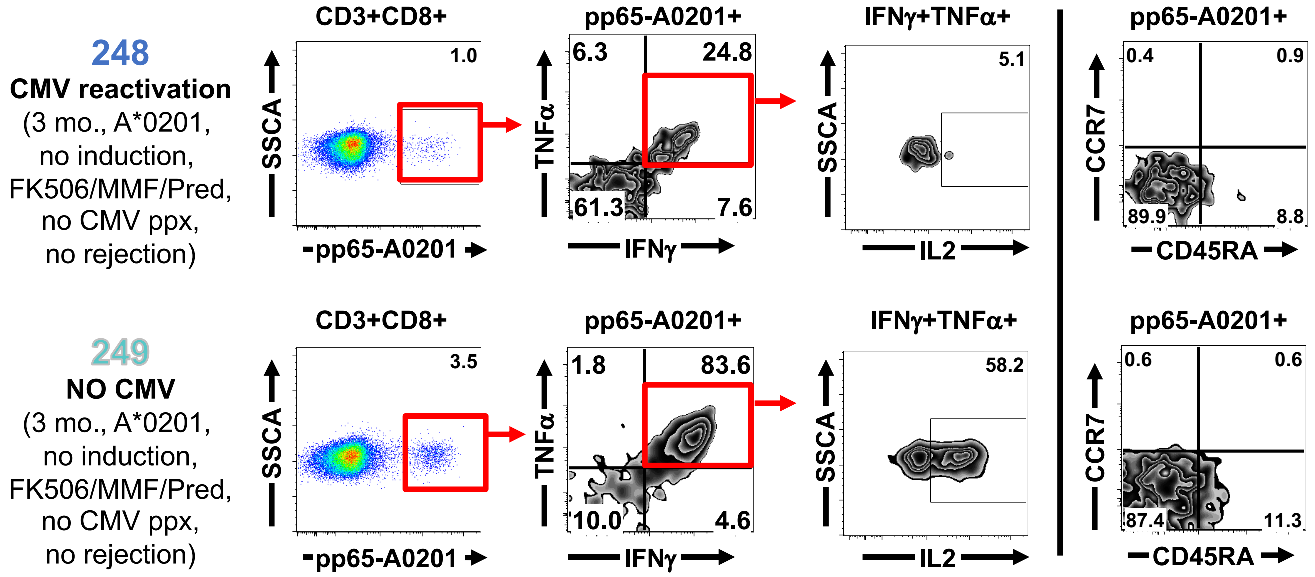

Supplemental Figure S14. Single-cell sorting and comparative cytokine and maturation staining of CMV pp65-HLA-A*0201 CD8+ T cells from a transplant recipient with (248, blue) and without (249, teal) CMV reactivation in the post-transplant period. Cryopreserved PBMC samples from two recipients were obtained from the Duke IRB-approved Abdominal Transplant Repository (ATR) (Pro00035555). Kidney, liver, pancreas, and small intestine transplant recipients were recruited prospectively through the Abdominal Transplant clinic at Duke University Hospital and PBMC samples were collected longitudinally at pre-specified time points prior to and following transplantation. One subject with and one matched control without CMV reactivation in the first 12 months following transplant were selected. The subjects were matched by age (50-55), HLA-*A*0201* status (necessary for tetramer use; note: no other matching alleles were required), type of transplant (kidney), induction immunosuppression (none), donor-recipient CMV status (D-/R+), maintenance immunosuppression (prednisone, mycophenolate (MMF), and tacrolimus (FK506)), and CMV prophylaxis (none). PBMC samples were selected from the time point just prior to when CMV reactivation occurred in the case subject (i.e., 3 months post-transplant for both the case and control subject). Five million cells were stimulated with the pp65-HLA-A*0201 dextramer labeled with PE and anti-CD28/anti-CD49 co-stimulation (1 μg/mL each). Following six hours of stimulation, cells were surface stained for viability, CD14, CD3, CD4, and CD8 and viable CD14-/CD3+/CD8+/pp65-HLA-A*0201 cells were sorted into a 96-well plate. Additionally, five million cells were stimulated in an analogous fashion, surface stained for phenotype, maturation, and antigen-exposure markers, then stained for cytokines via standard

intracellular staining protocol (BD CytoFix/CytoPerm). Despite drastic differences in cytokine expression and polyfunctionality between the two subjects, there was no difference in differentiation or antigen-exposure markers between the CMV-specific T cell populations.

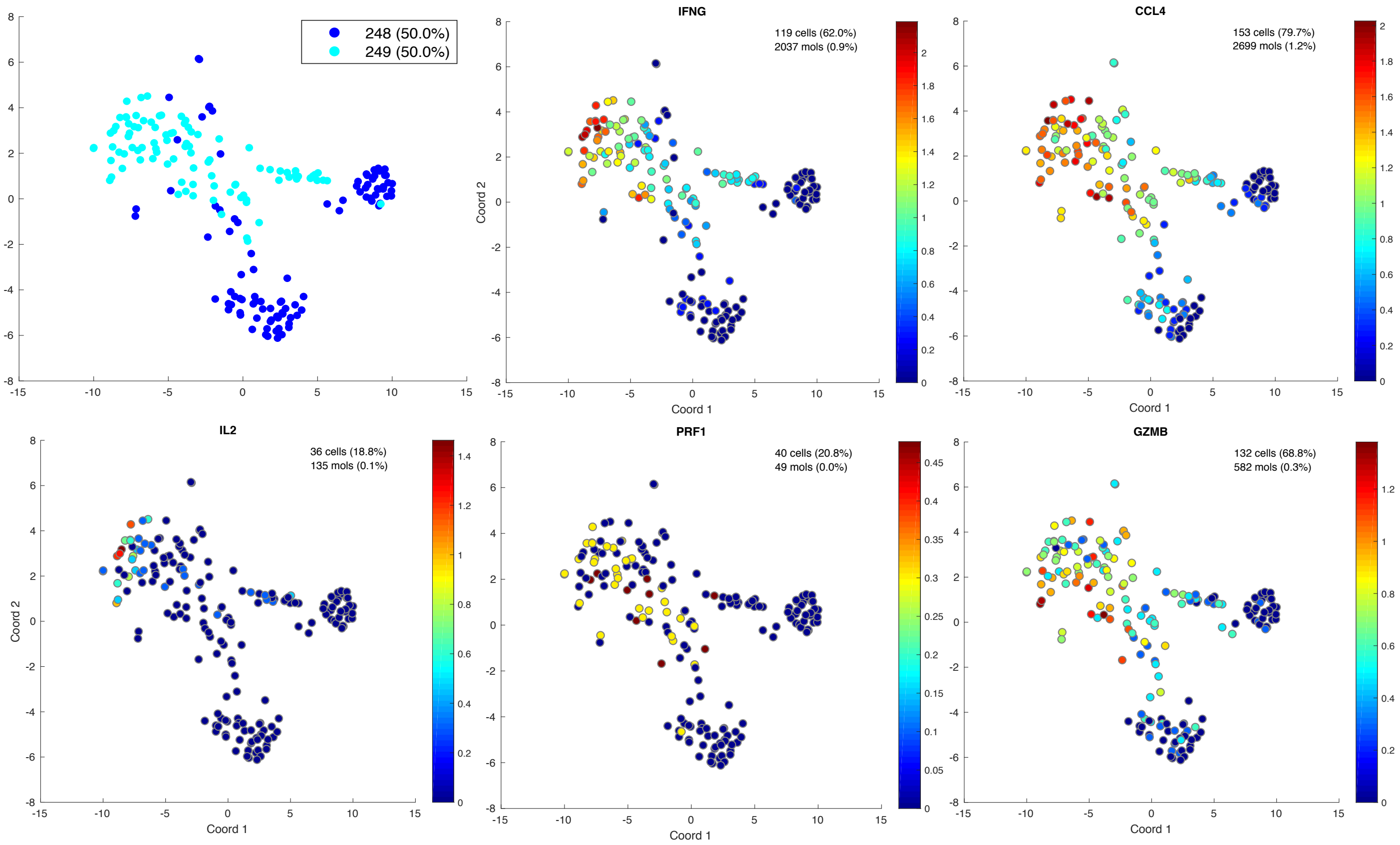

Supplemental Figure S15. PCA visualization of single cell data from the two transplant recipients. Absolute cytokine and chemokine (IFNγ, CCL4, IL-2, PRF1, GZMB) mRNA expression within these cell populations.

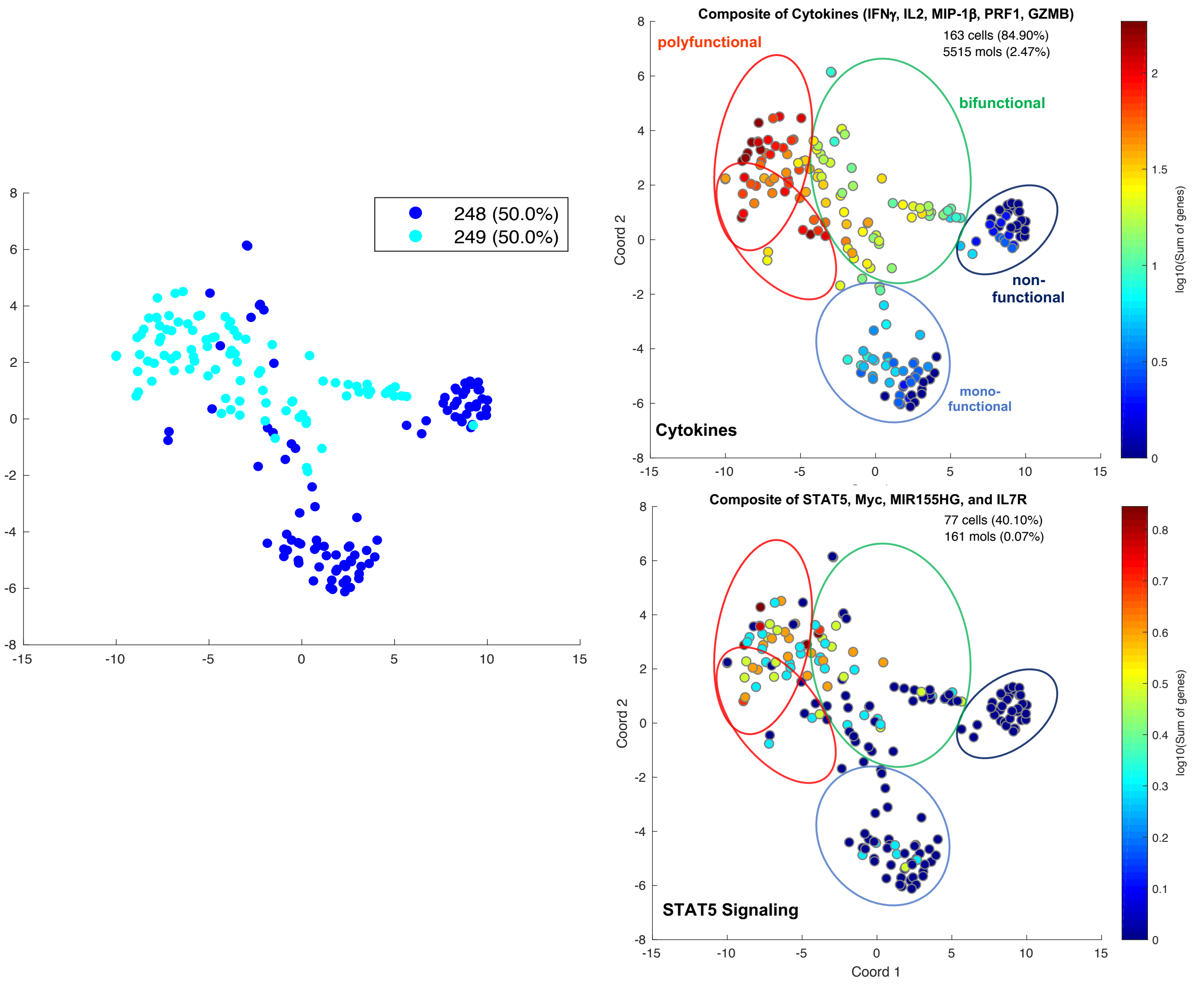

Supplemental Figure S16. PCA visualization of single cell data demonstrating relationship of functional subsets to the expression of STAT5-related genes (STAT5, myc, miR155HG, IL-7R, Bcl-2, Bcl-xL, Mcl-1, PIM2, Cyclin D1, etc).

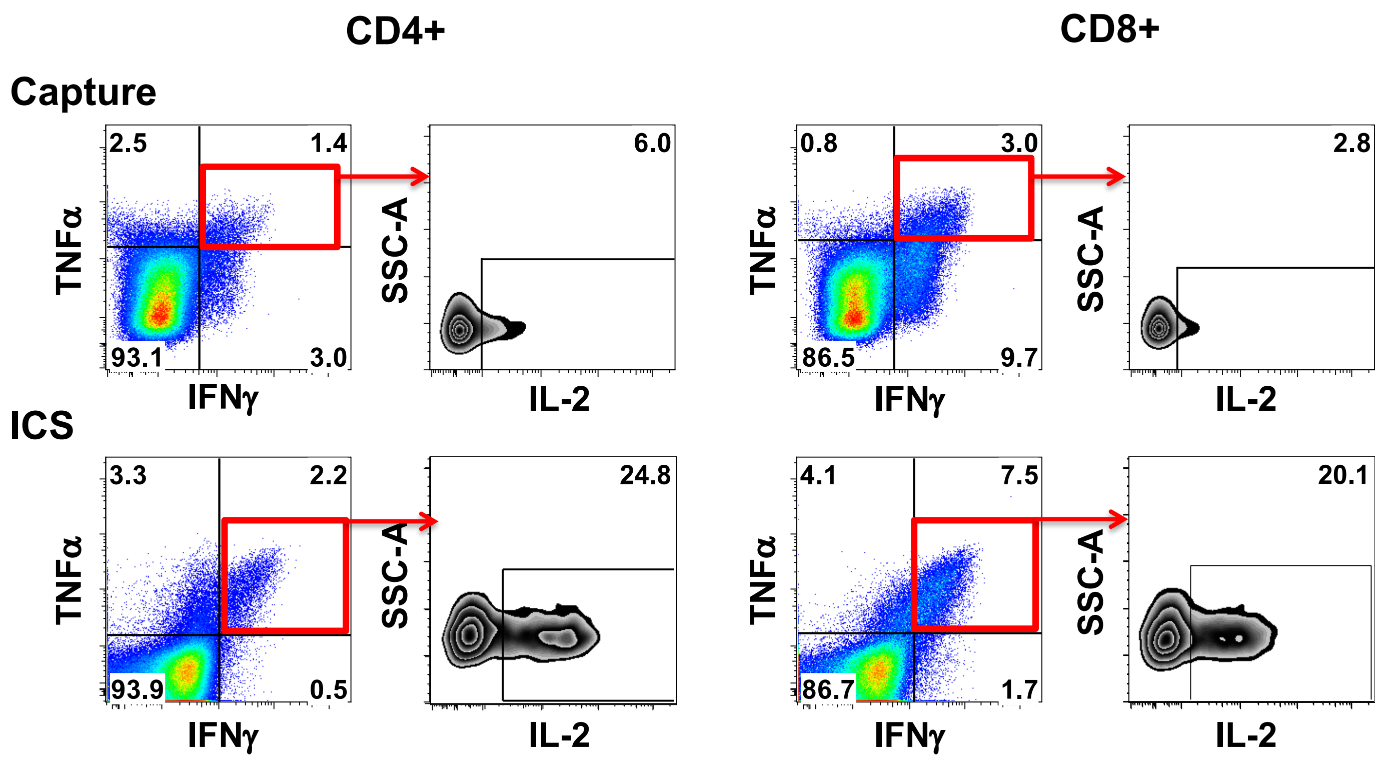

Supplemental Figure S17. Visual comparison of Miltenyi Cytokine Capture/Secretion assay and intracellular staining (ICS) of cytokines for quantification of CMV-specific functional subsets. In both the CD4+ and CD8+ populations, the Cytokine Capture assay using three independent cytokine targets yields reduced discrimination of polyfunctional T cells relative to ICS assay. Note the reduced resolution of bifunctional and polyfunctional T cell populations using the cytokine capture kit. This was difficult to further optimize due to manufacturer limitations regarding antibody-fluorophore conjugate availability for these kits. Additionally, the protocol requires that the cytokines of interest have a relatively similar expression profile (i.e., greater than or less than 5% of population) to allow for appropriate sample dilution during staining; while this prevents “bystander” staining, it can also lead to reduced staining efficacy for cytokines with low expression when paired with a highly-expressed cytokine. Finally, the cytokines must be simultaneously secreted during the same 45-minute period of time to ensure detection; current evidence suggests that cytokine secretion may in fact be sequential rather than simultaneous[3]. Additional information is provided in **Supplemental Table 2**.

SUPPLEMENTAL TABLES

**Table S1. Evidence supporting the role of antigen-specific polyfunctional T cells in immune response to pathogens, vaccination, and malignancy**
CMV reactivation and disease in:<br>• allogenic stem cell transplantation [4-13];<br>• solid organ transplant [14-18];<br>• liver transplantation [19];<br>• lung transplantation [20,21];<br>• kidney transplantation [22];<br>CMV in non-organ transplant immunocompromised subjects [23]<br>Vaccination [24-32].<br>HIV [33-43]<br>HCV [44]<br>Leishmaniasis<br>*T. cruzi* [45]<br>Toxoplasmosis [46]<br>*A. fumigatus*<br>*MTB* [47-50]<br>Flu [51]<br>EBV<br>BKV [52,53]<br>Cancer [54-60]

**Table S2. Advantages and Disadvantages of Cytokine Secretion/Capture and Intracellular Staining Assays**		
	Advantages	Disadvantages
Cytokine-secretion	• Allows for the isolation of living cells, which can then be used in a broad array of downstream assays, etc<br>• Increased yield and quality of RNA<br>• Reduced impact on downstream assays<br>• Utilizes clinical-grade reagents<br>• Cells may be isolated via magnetic-based techniques, which are more straightforward and cost-effective	• Limited to secreted molecules, and therefore not useful for transcription factors, phosphoproteins, etc.<br>• Risk of bystander labeling<br>• Requires *a priori* knowledge of expected level of cytokine production, which may become complicated when staining for multiple cytokines with highly variable expression levels<br>• Currently limited to one company, with limited number of fluorophores (FITC, APC, PE); therefore, only a limited number of cytokines may be assayed as one time.<br>• Requires dilutional step for labeling which may significantly alter signaling due to changes in antigen concentration<br>• Cells can't be fixed, therefore changes in transcription/translation, etc may occur during the time taken to process the samples before sorting/isolation<br>• When labeling multiple cytokines, they must be expressed simultaneously<br>• Capture of cytokines may significantly reduce cytokine-induced signaling<br>• Currently only available for cells expressing CD45
Intracellular Staining	• Allows for detection of cytokines as well as other intracellular proteins<br>• Fixation prevents further cellular processes, thus allowing one to capture the state of cell at a specific moment in time<br>• Ability to stain for numerous intracellular targets simultaneously (even up to 6 phosphoprotein targets)<br>• Does not require cytokines to be expressed simultaneously (although must be expressed within a 12-hour time-frame generally), nor to be expressed at relatively similar levels	• Cytokine assays require the use of protein transport inhibitors (e.g., brefeldin-A, monensin), which can be cytotoxic in a time- and cell-dependent manner<br>• Use of protein transport inhibitors also reduces cytokine secretion from the cell, potentially interfering with cytokine-induced signaling<br>• Fixation and permeabilization are required, which are associated with reduced quality of RNA and DNA, and significantly limits the ability to use samples for downstream assays<br>• Even with new, completely reversible methods of fixation, there is increased risk of loss of RNA and protein during permeabilization and cell labeling steps<br>• Requires flow cytometry-based cell sorting, which is labor-intensive and requires experienced operators.

SUPPLEMENTAL FIGURES/TABLES REFERENCES:

1. Illumina. Evaluating RNA quality from FFPE samples. Guidlines for obtaining high-quality RNA sequencing results from degraded RNA with the TruSeq(R) RNA Access Library Preparation Kit. 2014.

2. Opitz L, Tan G, Aquino C, Schlapbach R, Rehrauer H. Long Fragments Achieve Lower Base Quality in Illumina Paired-End Sequencing. J Biomol Tech 2019;30:S26.

3. Han Q, Bagheri N, Bradshaw EM, Hafler DA, Lauffenburger DA, Love JC. Polyfunctional responses by human T cells result from sequential release of cytokines. Proc Natl Acad Sci U S A 2012;109:1607-12.

4. Lilleri D, Fornara C, Chiesa A, Caldera D, Alessandrino EP, Gerna G. Human cytomegalovirus-specific CD4+ and CD8+ T-cell reconstitution in adult allogeneic hematopoietic stem cell transplant recipients and immune control of viral infection. Haematologica 2008;93:248-56.

5. Lilleri D, Gerna G, Fornara C, Lozza L, Maccario R, Locatelli F. Prospective simultaneous quantification of human cytomegalovirus-specific CD4+ and CD8+ T-cell reconstitution in young recipients of allogeneic hematopoietic stem cell transplants. Blood 2006;108:1406-12.

6. Krol L, Stuchly J, Hubacek P, et al. Signature profiles of CMV-specific T-cells in patients with CMV reactivation after hematopoietic SCT. Bone Marrow Transplant 2011;46:1089-98.

7. Munoz-Cobo B, Solano C, Benet I, et al. Functional profile of cytomegalovirus (CMV)-specific CD8+ T cells and kinetics of NKG2C+ NK cells associated with the resolution of CMV DNAemia in allogeneic stem cell transplant recipients. J Med Virol 2012;84:259-67.

8. Clari MA, Munoz-Cobo B, Solano C, et al. Performance of the QuantiFERON-cytomegalovirus (CMV) assay for detection and estimation of the magnitude and functionality of the CMV-specific gamma interferon-producing CD8(+) T-cell response in allogeneic stem cell transplant recipients. Clin Vaccine Immunol 2012;19:791-6.

9. Yong MK, Cameron PU, Slavin M, et al. Identifying Cytomegalovirus Complications Using the Quantiferon-CMV Assay After Allogeneic Hematopoietic Stem Cell Transplantation. J Infect Dis 2017;215:1684-94.

10. Yong MK, Lewin SR, Manuel O. Immune Monitoring for CMV in Transplantation. Curr Infect Dis Rep 2018;20:4.

11. Gimenez E, Blanco-Lobo P, Munoz-Cobo B, et al. Role of cytomegalovirus (CMV)-specific polyfunctional CD8+ T-cells and antibodies neutralizing virus epithelial infection in the control of CMV infection in an allogeneic stem-cell transplantation setting. J Gen Virol 2015;96:2822-31.

12. Gimenez E, Munoz-Cobo B, Solano C, et al. Functional patterns of cytomegalovirus (CMV) pp65 and immediate early-1-specific CD8(+) T cells that are associated with protection from and control of CMV DNAemia after allogeneic stem cell transplantation. Transpl Infect Dis 2015;17:361-70.

13. Camargo JF, Wieder E, Kimble E, et al. Deep functional immunophenotyping predicts risk of cytomegalovirus reactivation after hematopoietic cell transplantation. Blood 2018.

14. Gerna G, Lilleri D, Chiesa A, et al. Virologic and immunologic monitoring of cytomegalovirus to guide preemptive therapy in solid-organ transplantation. Am J Transplant 2011;11:2463-71.

15. Gerna G, Lilleri D, Fornara C, et al. Monitoring of human cytomegalovirus-specific CD4 and CD8 T-cell immunity in patients receiving solid organ transplantation. Am J Transplant 2006;6:2356-64.

16. Gabanti E, Bruno F, Lilleri D, et al. Human cytomegalovirus (HCMV)-specific CD4+ and CD8+ T cells are both required for prevention of HCMV disease in seropositive solid-organ transplant recipients. PLoS One 2014;9:e106044.

17. Lilleri D, Zelini P, Fornara C, et al. Human cytomegalovirus (HCMV)-specific T cell but not neutralizing or IgG binding antibody responses to glycoprotein complexes gB, gHgLgO, and pUL128L correlate with protection against high HCMV viral load reactivation in solid-organ transplant recipients. J Med Virol 2018;90:1620-8.

18. Lilleri D, Gerna G, Zelini P, et al. Monitoring of human cytomegalovirus and virus-specific T-cell response in young patients receiving allogeneic hematopoietic stem cell transplantation. PLoS One 2012;7:e41648.

19. Nebbia G, Mattes FM, Smith C, et al. Polyfunctional cytomegalovirus-specific CD4+ and pp65 CD8+ T cells protect against high-level replication after liver transplantation. Am J Transplant 2008;8:2590-9.

20. Snyder LD, Chan C, Kwon D, et al. Polyfunctional T-Cell Signatures to Predict Protection from Cytomegalovirus after Lung Transplantation. Am J Respir Crit Care Med 2016;193:78-85.

21. Snyder LD, Medinas R, Chan C, et al. Polyfunctional cytomegalovirus-specific immunity in lung transplant recipients receiving valganciclovir prophylaxis. Am J Transplant 2011;11:553-60.

22. Gasser O, Bihl F, Sanghavi S, et al. Treatment-dependent loss of polyfunctional CD8+ T-cell responses in HIV-infected kidney transplant recipients is associated with herpesvirus reactivation. Am J Transplant 2009;9:794-803.

23. Gibson L, Barysauskas CM, McManus M, et al. Reduced frequencies of polyfunctional CMV-specific T cell responses in infants with congenital CMV infection. J Clin Immunol 2015;35:289-301.

24. Saenz LA, Celis FP, Montoya CJ, Velilla PA. Polyfunctional CD8+ T-cell Response to Autologous Peptides from Protease and Reverse Transcriptase of HIV-1 Clade B. Curr HIV Res 2019.

25. L'Huillier AG, Ferreira VH, Hirzel C, et al. Cell-Mediated Immune Responses after Influenza Vaccination of Solid Organ Transplant Recipients: a Secondary Outcomes Analysis of a Randomized Controlled Trial. J Infect Dis 2019.

26. Valdes I, Lazo L, Hermida L, Guillen G, Gil L. Can Complementary Prime-Boost Immunization Strategies Be an Alternative and Promising Vaccine Approach Against Dengue Virus? Front Immunol 2019;10:1956.

27. Hessell AJ, Powell R, Jiang X, et al. Multimeric Epitope-Scaffold HIV Vaccines Target V1V2 and Differentially Tune Polyfunctional Antibody Responses. Cell Rep 2019;28:877-95 e6.

28. Tian Y, Babor M, Lane J, et al. Dengue-specific CD8+ T cell subsets display specialized transcriptomic and TCR profiles. J Clin Invest 2019;130:1727-41.

29. Lewinsohn DA, Lewinsohn DM, Scriba TJ. Polyfunctional CD4(+) T Cells As Targets for Tuberculosis Vaccination. Front Immunol 2017;8:1262.

30. Cohen KW, Frahm N. Current views on the potential for development of a HIV vaccine. Expert Opin Biol Ther 2017;17:295-303.

31. Maggioli MF, Palmer MV, Thacker TC, et al. Increased TNF-alpha/IFN-gamma/IL-2 and Decreased TNF-alpha/IFN-gamma Production by Central Memory T Cells Are Associated with Protective Responses against Bovine Tuberculosis Following BCG Vaccination. Front Immunol 2016;7:421.

32. Panagioti E, Redeker A, van Duikeren S, et al. The Breadth of Synthetic Long Peptide Vaccine-Induced CD8+ T Cell Responses Determines the Efficacy against Mouse Cytomegalovirus Infection. PLoS Pathog 2016;12:e1005895.

33. Akinsiku OT, Bansal A, Sabbaj S, Heath SL, Goepfert PA. Interleukin-2 production by polyfunctional HIV-1-specific CD8 T cells is associated with enhanced viral suppression. J Acquir Immune Defic Syndr 2011;58:132-40.

34. Betts MR, Harari A. Phenotype and function of protective T cell immune responses in HIV. Curr Opin HIV AIDS 2008;3:349-55.

35. Casetti R, De Simone G, Sacchi A, et al. Modulation of polyfunctional HIV-specific CD8 T cells in patients responding differently to antiretroviral therapy. Int J Immunopathol Pharmacol 2014;27:291-7.

36. Daucher M, Price DA, Brenchley JM, et al. Virological outcome after structured interruption of antiretroviral therapy for human immunodeficiency virus infection is associated with the functional profile of virus-specific CD8+ T cells. J Virol 2008;82:4102-14.

37. Erickson AL, Willberg CB, McMahan V, et al. Potentially exposed but uninfected individuals produce cytotoxic and polyfunctional human immunodeficiency virus type 1-specific CD8(+) T-cell responses which can be defined to the epitope level. Clin Vaccine Immunol 2008;15:1745-8.

38. Lelic A, Verschoor CP, Ventresca M, et al. The polyfunctionality of human memory CD8+ T cells elicited by acute and chronic virus infections is not influenced by age. PLoS Pathog 2012;8:e1003076.

39. Makedonas G, Betts MR. Polyfunctional analysis of human t cell responses: importance in vaccine immunogenicity and natural infection. Springer Semin Immunopathol 2006;28:209-19.

40. Rehr M, Cahenzli J, Haas A, et al. Emergence of polyfunctional CD8+ T cells after prolonged suppression of human immunodeficiency virus replication by antiretroviral therapy. J Virol 2008;82:3391-404.

41. Riou C, Burgers WA, Mlisana K, et al. Differential impact of magnitude, polyfunctional capacity, and specificity of HIV-specific CD8+ T cell responses on HIV set point. J Virol 2014;88:1819-24.

42. Techakriengkrai N, Tansiri Y, Hansasuta P. Poor HIV control in HLA-B*27 and B*57/58 noncontrollers is associated with limited number of polyfunctional Gag p24-specific CD8+ T cells. AIDS 2013;27:17-27.

43. Van Braeckel E, Desombere I, Clement F, et al. Polyfunctional CD4(+) T cell responses in HIV-1-infected viral controllers compared with those in healthy recipients of an adjuvanted polyprotein HIV-1 vaccine. Vaccine 2013;31:3739-46.

44. Ciuffreda D, Comte D, Cavassini M, et al. Polyfunctional HCV-specific T-cell responses are associated with effective control of HCV replication. Eur J Immunol 2008;38:2665-77.

45. Albareda MC, De Rissio AM, Tomas G, et al. Polyfunctional T cell responses in children in early stages of chronic Trypanosoma cruzi infection contrast with monofunctional responses of long-term infected adults. PLoS Negl Trop Dis 2013;7:e2575.

46. Bhadra R, Gigley JP, Khan IA. PD-1-mediated attrition of polyfunctional memory CD8+ T cells in chronic toxoplasma infection. J Infect Dis 2012;206:125-34.

47. Burel JG, Apte SH, Groves PL, McCarthy JS, Doolan DL. Polyfunctional and IFN-gamma monofunctional human CD4(+) T cell populations are molecularly distinct. JCI Insight 2017;2:e87499.

48. Counoupas C, Pinto R, Nagalingam G, Britton WJ, Petrovsky N, Triccas JA. Delta inulin-based adjuvants promote the generation of polyfunctional CD4(+) T cell responses and protection against Mycobacterium tuberculosis infection. Sci Rep 2017;7:8582.

49. Smith SG, Zelmer A, Blitz R, Fletcher HA, Dockrell HM. Polyfunctional CD4 T-cells correlate with in vitro mycobacterial growth inhibition following Mycobacterium bovis BCG-vaccination of infants. Vaccine 2016;34:5298-305.

50. Sutherland JS, Young JM, Peterson KL, et al. Polyfunctional CD4(+) and CD8(+) T cell responses to tuberculosis antigens in HIV-1-infected patients before and after anti-retroviral treatment. J Immunol 2010;184:6537-44.

51. Makedonas G, Hutnick N, Haney D, et al. Perforin and IL-2 upregulation define qualitative differences among highly functional virus-specific human CD8 T cells. PLoS Pathog 2010;6:e1000798.

52. Blyth E, Clancy L, Simms R, et al. BK virus-specific T cells for use in cellular therapy show specificity to multiple antigens and polyfunctional cytokine responses. Transplantation 2011;92:1077-84.

53. Schaenman JM, Korin Y, Sidwell T, et al. Increased Frequency of BK Virus-Specific Polyfunctional CD8+ T Cells Predict Successful Control of BK Viremia After Kidney Transplantation. Transplantation 2017;101:1479-87.

54. Ding ZC, Blazar BR, Mellor AL, Munn DH, Zhou G. Chemotherapy rescues tumor-driven aberrant CD4+ T-cell differentiation and restores an activated polyfunctional helper phenotype. Blood 2010;115:2397-406.

55. Ding ZC, Habtetsion T, Cao Y, et al. Adjuvant IL-7 potentiates adoptive T cell therapy by amplifying and sustaining polyfunctional antitumor CD4+ T cells. Sci Rep 2017;7:12168.

56. Ding ZC, Huang L, Blazar BR, et al. Polyfunctional CD4(+) T cells are essential for eradicating advanced B-cell lymphoma after chemotherapy. Blood 2012;120:2229-39.

57. Ding ZC, Liu C, Cao Y, et al. IL-7 signaling imparts polyfunctionality and stemness potential to CD4(+) T cells. Oncoimmunology 2016;5:e1171445.

58. Mousset CM, Hobo W, Ji Y, et al. Ex vivo AKT-inhibition facilitates generation of polyfunctional stem cell memory-like CD8(+) T cells for adoptive immunotherapy. Oncoimmunology 2018;7:e1488565.

59. Tran E, Nielsen JS, Wick DA, et al. Polyfunctional T-cell responses are disrupted by the ovarian cancer ascites environment and only partially restored by clinically relevant cytokines. PLoS One 2010;5:e15625.

60. Xue Q, Bettini E, Paczkowski P, et al. Single-cell multiplexed cytokine profiling of CD19 CAR-T cells reveals a diverse landscape of polyfunctional antigen-specific response. J Immunother Cancer 2017;5:85.
